# Honorary authorship is highly prevalent in health sciences: systematic review and meta-analysis of surveys

**DOI:** 10.1038/s41598-024-54909-w

**Published:** 2024-02-22

**Authors:** Reint A. Meursinge Reynders, Gerben ter Riet, Nicola Di Girolamo, Davide Cavagnetto, Mario Malički

**Affiliations:** 1 Department of Oral and Maxillofacial Surgery, Amsterdam University Medical Center (Amsterdam UMC) Location AMC, Meibergdreef 9, 1105 AZ Amsterdam, The Netherlands; 2 Studio di Ortodonzia, Via Matteo Bandello 15, 20123 Milan, Italy; 3 Urban Vitality Centre of Expertise, Amsterdam University of Applied Sciences, Amsterdam, The Netherlands; 4 Department of Cardiology, Amsterdam University Medical Center (Amsterdam UMC) Location AMC, Meibergdreef 9, 1105 AZ Amsterdam, The Netherlands; 5 Department of Clinical Sciences, College of Veterinary Medicine, Cornell University, 930 Campus Rd, Ithaca, NY 14853 USA; 6 EBMVet, Via Sigismondo Trecchi 20, 26100 Cremona, CR Italy; 7 Stanford Program on Research Rigor and Reproducibility (SPORR), Stanford University, Stanford, CA USA; 8 Department of Epidemiology and Population Health, Stanford University, Stanford, CA USA; 9 Meta-Research Innovation Center at Stanford (METRICS), Stanford University, Stanford, CA USA

**Keywords:** Health care, Medical research

## Abstract

A systematic review and meta-analysis of survey research was conducted to estimate honorary authorship prevalence in health sciences. We searched PubMed, Lens.org, and Dimensions.ai. until January 5 2023. Methodological quality was assessed and quantitative syntheses were conducted. Nineteen surveys were included and rated as having low methodological quality. We found a pooled prevalence of 26% [95% CI 21–31] (6 surveys, 2758 respondents) of researchers that perceived co-author(s) as honorary on the publication at issue (when they were not referred to any authorship criteria). That prevalence was 18% [95% CI 15–21] (11 surveys, 4272 respondents) when researchers were referred to Committee of Medical Journal Editors (ICMJE) authorship criteria, and 51% [95% CI 47–56] (15 surveys, 5111 respondents) when researchers were asked to declare their co-author(s) contributions on the publication at issue (and these were then compared to ICMJE criteria). 10% of researchers [95% CI 9–12] (11 surveys, 3,663 respondents) reported being approached by others to include honorary author(s) on the publication at issue and 16% [95% CI 13–18] (2 surveys, 823 respondents) admitted adding (an) honorary author(s). Survey research consistently indicates that honorary authorship in the health sciences is highly prevalent, however the quality of the surveys’ methods and reporting needs improvement.

## Introduction

Authorship in scientific publications documents research output and is conducive to one’s career^1–3^. Authorship was labeled as one of the major currencies in science^4^. Honorary authorship (HA) refers to authorship assigned to individuals that should not have been included as authors of a publication, because they made no or insufficient contributions to qualify as authors^5^. HA can occur when there is little external control, when major power imbalances between researchers exist, but also absent of those imbalances. To limit the inappropriate assignment of authorship and to reduce authorship disputes, various organizations have developed authorship guidelines^6–8^. Such guidelines are essential, but without their adoption by the pertinent journals, the practice is likely to continue^9^. The criteria for authorship developed by the International Committee of Medical Journal Editors (ICMJE)^7^ are the most well-known guidelines in the health sciences and are often used to assess if an honorary author was listed in a particular publication^10–12^. The ICMJE criteria have been modified over the years^13^, still some consider them too restrictive^8^ and their wording such as, ‘substantial’, ‘critically’, ‘important’, and ‘appropriately’ are prone to subjective interpretation.

Surveys may shed light on HA prevalence and its potential drivers and when repeated, they may be used to monitor the effects, if any, of initiatives aimed to reduce HA. We assessed HA prevalence in health sciences using five prespecified HA operationalizations. These five operationalizations all pertain to single target publications on which surveyees were questioned (Review items 1–5):

1. Researchers perceiving other co-author(s) as honorary author(s) on a publication.
2. Researchers having been approached by others to include honorary author(s) on a publication.
3. Researchers admitting being an honorary author on a publication.
4. Researchers admitting adding honorary author(s) on a publication.
5. Researchers admitting having approached others to include honorary author(s) on a publication.

Per our protocol, items review 1 and 2 were our primary objective, and 3–5 secondary. In addition, we planned: (a) to look at all five objectives separately when no authorship criteria were disclosed to survey respondents, e.g., based on the personal perception/opinion of the surveyee and when ICMJE-criteria were disclosed; and (b) to check for HA prevalence changes over time^5^. All the objectives were planned to be assessed only in surveys that asked for HA in one specific publication (e.g., researchers last publication), and not in surveys asking if researchers ever in their lifetime (all scholarly corpus) had at least one HA.

## Methods

We reported the study according to the Preferred Reporting Items for Systematic review and Meta-Analysis (PRISMA)^14,15^. Table 1 lists key terminology^5,7,16,17^. The protocol for this systematic review was registered in the Open Science Framework (OSF) (https://osf.io/5nvar/) peer reviewed and published^5^. In this section we briefly report on the review methods. Details can be found in the protocol^5^ and Appendix. Differences between the protocol and the completed systematic review are explained in the (Appendix. Additional item A, pages 2 and 3). The main difference was omitting double arcsine transformation prior to statistical pooling, because recent work has shown it to be invalid in meta-analysis of proportions^18,19^.

**Table 1:** Glossary of terms.

Term	Definition
Survey^16^	Wikipedia^16^ defines a survey as follows: ‘In research of human subjects, a survey is a list of questions aimed for extracting specific data from a particular group of people’
Surveyee	Any author on the author list of a scientific publication, e.g., first, last, corresponding author, that was invited to participate in a survey on at least one of our review items
Health sciences^17^	Wikipedia^17^ defines ‘health sciences’ as: ‘are those sciences which focus on health, or health care, as core parts of their subject matter. Health sciences relate to multiple academic disciplines, including STEM disciplines and emerging patient safety disciplines (such as social care research)
Honorary authorship^5^	Refers to authorship assigned to individuals that should not have been included as authors of a publication, because they made no or insufficient contributions to qualify as authors
ICMJE-defined criteria for authorship^7^	The ICMJE^7^ recommends that authorship is based on the following 4 criteria: (1) ‘Substantial contributions to the conception or design of the work; or the acquisition, analysis, or interpretation of data for the work; AND (2) Drafting the work or revising it critically for important intellectual content; AND (3) Final approval of the version to be published; AND (4) Agreement to be accountable for all aspects of the work in ensuring that questions related to the accuracy or integrity of any part of the work are appropriately investigated and resolved.’
ICMJE-based honorary authorship	The perception/opinion of the surveyee that one or more of the co-authors did not meet the criteria for authorship of the ICMJE
Perceived honorary authorship	The perception/opinion of the surveyee that one or more of the co-authors should not have been included as author(s) of a publication, because they made no or insufficient contributions to qualify as authors

### Eligibility criteria

We included publications in health sciences which reported on results of surveys in any language, and in any setting and at any time point on a series of pre-defined authorship issues in one particular publication selected by the survey authors, i.e., the publication at issue^20,21^ (Appendix. Additional item B, page 4).

### Information sources and search strategy

PubMed, Lens.org, and Dimensions.ai were searched for eligible surveys from inception until January 5 2023. No language or date filters were applied, except health sciences filters for the full search strategies for Lens.org and Dimensions.ai. References of the included studies were also searched for additional eligible surveys. Search strategies were piloted and can be found in the protocol^5^ (Appendix. Additional item C, page 5).

### Survey selection process and data collection

The publication screening and data collection procedures were conducted by two authors (RMR and DC) independently. Disagreements were resolved through discussions, by contacting authors to obtain additional information, and through arbitration by a third author (GTR). Rayyan^22^ was used for title and abstract screening. Full texts of potentially eligible surveys were retrieved and assessed for eligibility. References of eligible studies were screened for additional eligible surveys. We report a list of all manuscripts that were initially selected for full text screening, but were excluded with rationale for doing so. All data to be extracted from the eligible surveys were collected in pilot-tested data forms (Appendix. Additional item D, pages 6–10).

### Study risk of bias assessment

We developed a critical appraisal tool with a 14 items checklist to assess the methodological quality of each eligible result of each included survey^5^ (Appendix. Additional item E, pages 11 and 12). We also assessed how the non-implementation of these quality safeguards could have affected the results of the survey. In line with the AMSTAR-2 tool^23^ we labeled 7 of the 14 items as ‘critical’, due to their large influence on the validity of results. We adopted the 4 ratings of the AMSTAR-2 tool, i.e., ‘high’, ‘moderate’, ‘low’, and ‘critically low’ to rate the overall confidence for each assessed result. The Appendix (Additional item E, page 14) has details of ratings. All assessments and ratings were done by 2 reviewers (RMR and DC) independently and disagreements were resolved as stated above. Additional information on development and use of the critical appraisal tool is in the protocol^5^ and in the Appendix (Additional item E, pages 11–14).

### Occurrence measures and synthesis methods

Prevalence was the occurrence measure used in the quantitative syntheses and in the presentation of single outcomes. Prevalences were given with their exact (Wilson) 95% confidence intervals. For each outcome we measured the prevalence as defined in our objectives. We also reported the response rates in each eligible survey. The definitions of all outcomes and the respective numerators and denominators followed our protocol (Appendix Table A15, page 15). The (target) publication on which the surveyee was surveyed was the unit of analysis for the 5 review items, i.e., only occurrences of HA issues in the specific publication singled out by the surveyors were assessed, and not for example occurrences of HA issues in all manuscripts a surveyee had ever published.

We first conducted a systematic narrative synthesis for all outcomes. Our protocol and the Appendix Additional item F, page 16) list the criteria for precluding meta-analyses. We pooled prevalences and displayed them in forest plots with their 95% confidence intervals using random effects models. The metaprop command in Stata 18 was used to perform the statistics^24^. We checked whether respondents participated in one survey more than once, i.e., whether surveyees had published multiple articles in the eligible time span and were asked to submit a questionnaire for each published article (Appendix Table A6, page 7). To deal with missing data we contacted the corresponding author or a co-author if the corresponding author did not reply within 2 weeks. Authors were contacted by email and 2 reminders were sent, one and two weeks after the initial email, respectively. When no response was received the data were coded as missing.

### Investigation of heterogeneity and sensitivity analyses

We assessed the presence and extent of heterogeneity by visually inspecting the overlap of the confidence intervals in the forest plots, conducting the test of homogeneity (Chi^2^), and calculating the estimate of between study variance (tau^2^) and *I*^2^ to measure the inconsistency in the results^25^. We also sought explanations for diversity through meta-regression and subgroup analyses of both survey-and methodology-related explanatory variables^5^ (Appendix, Additional item F, page 16). We also considered specific issues to explore in sensitivity analyses, e.g., the impact of the quality or the characteristics of the survey design of certain reviews on the results of this systematic review^5^.

### (Non) reporting bias assessment

We adopted the term non-reporting bias over reporting bias as suggested by Cochrane^26^. We used various strategies to address non-reporting biases as reported in our protocol and Appendix (Additional item G, page 17). We did not conduct tests for funnel plot asymmetry, because there is no evidence that proportional data adequately adjust for these graphical tests^27^.

### Certainty assessment

We used the GRADE approach to assess the overall certainty of the body of evidence^28^. According to GRADE we assigned four levels of certainty: ‘high’, ‘moderate’, ‘low’, and ‘very low certainty’^28^. We presented the GRADE ratings for each outcome together with our rationales in a summary of findings table. Guidance for grading the certainty of evidence for a review item is in the Appendix (Additional item H, pages 18 and 19).

## Results

### Study selection and study characteristics

**Figure 1:**
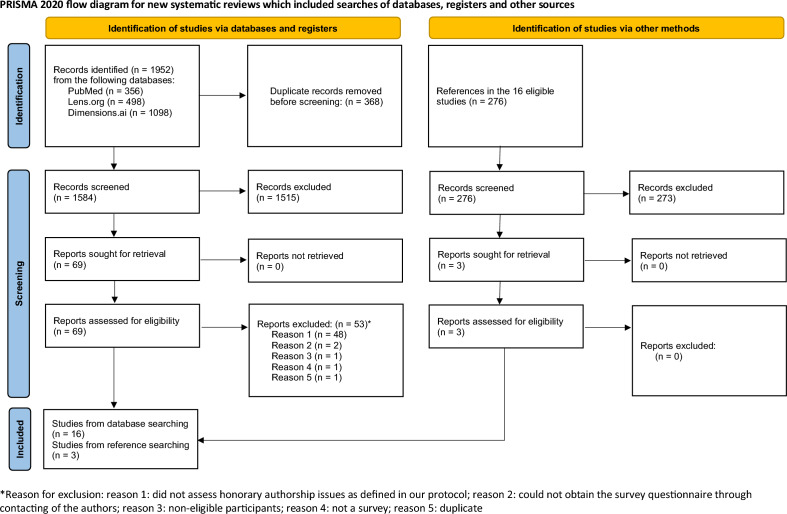
PRISMA flow diagram.

Figure 1 shows the study flow^14,15^. Our searches identified 1952 records. After deduplication, 1584 records remained for screening and 16 were eligible. The references of these 16 surveys provided another 3 eligible articles making 19 eligible surveys in total, which used 51 eligible questions to assess the prevalence of HA issues. The Appendix (Additional item I, page 20)shows all included surveys and full text reports that were assessed for eligibility and that were subsequently excluded with the rationales for exclusion (Appendix. Additional item J, pages 25–28). We contacted the authors of 15 of the 19 eligible surveys either to verify eligibility or to obtain missing information. The questionnaires for all 19 included surveys were reported in the pertinent manuscripts or obtained via the authors. Complete gender/sex breakdowns for all considered categories were not implemented in any of the included surveys and only the terms ‘males’ and ‘females’ were used^29^. Males were the predominant respondents (> 51%) in the 13 of 14 surveys that reported the sex distribution among respondents. The percentage of surveyees that were an associate professor or higher was at least 30% in 10 of 11 surveys reporting this proportion. The prevalences of the countries or continents of origin of surveyees could not be reliably extracted, because of imprecise, partial or non-reporting of this information. The Appendix (Additional item I, pages 21–24) shows additional characteristics of the 19 surveys.

### Assessment of methodological quality

The overall confidence in 24 results was rated as ‘critically low’ and ‘low’ for 27 results, i.e., based on the 14-item quality checklist^5^. Table 2 shows the 7 items of this checklist that we considered ‘critical’ alongside the frequency of their ratings^30^. The characteristics of responding surveyees and the review items were defined for all 51 questions (items 6 and 8). However, it was unclear whether the respondents were representative of the target population due to poor reporting on socio-demographics, response rates, and the intended target population. Further, various quality safeguards were not implemented and could have affected the results, i.e., limitations in the survey methods for 37% (19/51) of results, low response rates or weighting issues in most results (86% (44/51), and inadequate sample sizes for 35% (18/51) of results. The ratings for each of the 7 critical items of the 51 results are given in the Appendix (Additional item K, pages 29–32).

**Table 2:** Prevalence of answers to the seven critical items of the quality checklist.

Quality checklist question (item number)	Unclear*	Yes	No
Was there selective (non) reporting regarding the review item (#)? (Item 2)	100% (51/51)	0%	0%
Were there no survey methods that could have introduced bias? (Item 5)	63% (32/51)	0%	37% (19/51)
Were the characteristics of the responding surveyees on the review item defined? (Item 6)	0%	100% (51/51)	0%
Were the characteristics of the responding surveyees on the review item representative of the target population (Item 7)	100% (51/51)	0%	0%
Was the review item defined? (Item 8)	0%	100% (51/51)	0%
Did the magnitude of the response rate on the review item or the way the response rate (in the case of a low response rate) was managed provide certainty in the validity of the results on the review item (Item 12)	0%	14% (7/51)	86% (44/51)
Was the sample size adequate for the prevalence statistic of the review item? (Item 13)**	0%	65% (33/51)	35% (18/51)

*’Unclear’ was assigned when too few details were reported in the manuscript or additional files to make a judgment of assigning ‘Yes’ or ‘No’.

** the required sample size was calculated with EpiTools epidemiological calculators and was based on the identified prevalence and the total sample size^29^. The estimated prevalence was calculated with a 0.95 confidence level (desired precision of estimate 0.05).

### Response rates

All response rates except 2^31,32^, of the 19 eligible surveys were below 50% (Tables 3 and 4) (Appendix, Additional item L, pages 33–36). Three types of denominators were used to calculate these rates: N1: Number of emails with questionnaires sent, N2: Number of emails with questionnaires not bounced, N3: Number of emails with questionnaires for which the surveyee was available, which implies for example ‘not on vacation’, ‘not on strike’, ‘not on maternity leave’ etc. We prefer N2 or N3 over N1, but often only N1 was given. In Tables 3 and 4 we report which denominator was used to calculate response rates. When the same denominators were used, we synthesized these rates quantitatively using the random effects model (Tables 3 and 4) (Appendix, Additional item L, pages 33–36).

**Table 3:** Survey response rates and results. Part 1.

Review items (objectives) and questions they were assessed with in published surveys	Response rate	Number of surveys	Prevalence of review item
Review Item 1. Co-author(s) as honorary author(s) on a publication			
**Review item 1a.** Perceived honorary authorship of a co-author (without researchers being referred to any specific criteria for authorship) **Question 1a.** Do you feel that any of your co-authors in this article did not make sufficient contributions to merit being included as co-authors?	29% (392/1,338) (N1*) [95% CI 27–32] (n = 1338 surveyees)(1 survey) 22% (N2*) [95% CI 15–30] (n = 10,154 surveyees)(3 surveys) 19% (N3*) [95% CI 18–21] (n = 2747 surveyees)(2 surveys)	N = 6	26% [95% CI 21–31] (n = 2758 respondents)
**Review item 1b.** Perceived honorary authorship based on ICMJE criteria **Question 1b**. As a result of your current understanding of ICMJE authorship guidelines, do you believe that any of your coauthors listed for this article did not make sufficient contributions to merit being included as coauthors?	22% (N1*) [95% CI 19–26] (n = 4098 surveyees)(3 surveys) 31% (N2*) [95% CI 25–37] (n = 5808 surveyees)(5 surveys) 41% (N3*) [95% CI 27–55] (n = 3769 surveyees)(3 surveys)	N = 11	18% [95% CI 15–21] (n = 4272 respondents)
**Review item 1c.** Honorary authorship based on researchers’ reported contributions of co-authors compared to the ICMJE criteria **Question 1c.** **Did any of your co-authors perform only one or more of the following tasks, and nothing else, while working on this article? These tasks refer to: 1. Supervising/recruiting co-authors 2. Obtaining funding or material support 3. Recruiting study subjects 4. Performing cases used in the study 5. Contributing illustrations 6. Reviewing the manuscript 7. Approving manuscript before submission to a journal 8. Signing statement of copyright transfer to journal	24% (N1*) [95% CI 20–28] (n = 5436 surveyees)(4 surveys) 28% (N2*) [95% CI 23–33] (n = 11,837 surveyees)(10 surveys) 39% (585/1511) (N3*) [95% CI 36–41] (n = 1511 surveyees)(1 survey)	N = 15	51% [95% CI 47–56] (n = 5111 respondents)
** Review item 1c.** Honorary authorship based on researchers’ reported contributions of co-authors compared to the ICMJE criteria ** Question 1d.** ** How many of your coauthors had only one of the following functions, meaning they did only one of these functions and nothing else? (For each function below, give the number of coauthors, who did only that function) These functions refer to: 1. Conceiving or designing the work 2. Conducting the literature search 3. Analyzing/interpreting literature/data 4. Performing statistical analysis 5. Writing the manuscript or part of the manuscript 6. Revising the manuscript critically for important intellectual content 7. Approving manuscript before submission to a journal 8. Supervising the work or any of the coauthors 9. Recruiting coauthors 10. Communicating with journal editor(s) 11. Obtaining funding or material support 12. Reviewing proofs or the journal's edited version of the review	54.5% (666/1221)(N3*) [95% CI 52–57] (n = 1221 surveyees)(1 survey)	N = 1 (Gülen 2020)	22.2% (148/666) [95% CI 19–26] (n = 666 respondents)
Review item 2. Researchers having been approached by others to include honorary author(s) on a publication			
** Review item 2.** Researchers having been approached by others to include honorary author(s) on a publication ** Question 2.** ***Did anyone suggest that you include an 'honorary' author in your manuscript?	20% (N1*) [95% CI 19–22] (n = 2,739 surveyees)(2 surveys) 26% (N2*) [95% CI 22–31] (n = 9,750 surveyees)(8 surveys) 38.6% (583/1511) (N3*) [95% CI 36–41] (n = 1511 surveyees)(1 survey)	N = 11	10% [95% CI 9–12] (n = 3,663 respondents)

*N1: Number of emails with questionnaires sent, N2: Number of emails with questionnaires not bounced, N3: Number of questionnaires for which the surveyee was available. **International Committee of Medical Journal Editors (ICMJE)-based honorary authorship. *** Not specified whether it was perceived or ICMJE-based honorary authorship or both.

**Table 4:** Survey response rates and results. Part 2.

Review items (objectives) and questions they were assessed with in published surveys	Response rate	Number of surveys	Prevalence of review item
Review item 3. Researchers admitting being an honorary author(s) on a publication			
**Review item 3a**. Researchers admitting being an honorary author(s) on a publication based on a list of author’s contributions. First author did not conceive or design the work, conduct literature search, or analyze and interpret data **Question 3a.** ** Think about your role in the development of the review. Check all of the functions you personally performed for the review. Functions: 1. Conceiving or designing the work 2. Conducting the literature search 3. Analyzing/interpreting literature/data 4. Performing statistical analysis 5. Writing the manuscript or part of the manuscript 6. Revising the manuscript critically for important intellectual content 7. Approving manuscript before submission to a journal 8. Supervising the work or any of the coauthors 9. Recruiting coauthors 10. Communicating with journal editor(s) 11. Obtaining funding or material support 12. Reviewing proofs or the journal's edited version of the review	54.5% (666/1,221) (N3)* [95% CI 51.7–57.3] (n = 1221 surveyees)	N = 1 (Gülen 2020)^30^	0.15% (1/666) [95% CI 0.0038–0.83] (n = 666 respondents)
**Review item 3b**. Researchers admitting being an honorary author(s) on a publication based on a list of author’s contributions. First author did not draft or revise the review **Question 3b.** ** Think about your role in the development of the review. Check all of the functions you personally performed for the review. Functions: 1. Conceiving or designing the work 2. Conducting the literature search 3. Analyzing/interpreting literature/data 4. Performing statistical analysis 5. Writing the manuscript or part of the manuscript 6. Revising the manuscript critically for important intellectual content 7. Approving manuscript before submission to a journal 8. Supervising the work or any of the coauthors 9. Recruiting coauthors 10. Communicating with journal editor(s) 11. Obtaining funding or material support 12. Reviewing proofs or the journal's edited version of the review	54.5% (666/1,221) (N3)* [95% CI 51.7–57.3] (n = 1221 surveyees)	N = 1 (Gülen 2020)^30^	0.15% (1/666) [95% CI 0.0038–0.83] (n = 666 respondents)
**Review item 3c**. Researchers admitting being an honorary author(s) on a publication based on a list of author’s contributions. First author did not give the final approval **Question 3c.** ** Think about your role in the development of the review. Check all of the functions you personally performed for the review. Functions: 1. Conceiving or designing the work 2. Conducting the literature search 3. Analyzing/interpreting literature/data 4. Performing statistical analysis 5. Writing the manuscript or part of the manuscript 6. Revising the manuscript critically for important intellectual content 7. Approving manuscript before submission to a journal 8. Supervising the work or any of the coauthors 9. Recruiting coauthors 10. Communicating with journal editor(s) 11. Obtaining funding or material support 12. Reviewing proofs or the journal's edited version of the review	54.5% (666/1,221) (N3)* [95% CI 51.7–57.3] (n = 1221 surveyees)	N = 1 (Gülen 2020)^30^	7.2% (48/666) [95% CI 5.4–9.4] (n = 666 respondents)
** Review item 3d**. Researchers admitting being an honorary author(s) on a publication based on a list of author’s contributions ** Question 3d.** ** In the spaces marked "Contribution Codes," those code letters from the box should be marked that designate substantive contribution(s) of individual authors to the paper. These contribution codes refer to: 1. Conception and design 2. Analysis and interpretation of the data 3. Provision of study materials or patients 4. Collection, assembly and possession of raw data 5. Statistical expertise 6. Drafting of the article 7. Critical revision of the article for important intellectual content 8. Final approval of the article 9. Obtaining of funding 10. Administrative, technical, or logistic support 11. Guarantor of the study 12. Other (specify):	72% (201/279) (N1)* [95% CI 66.4–77.2] (n = 279 surveyees)	N = 1 (Ilakovac 2007)^31^	33.3% (67/201) [95% CI 26.9–40.3] (n = 201 respondents)
Review item 4. Researchers admitting adding an honorary author(s) on a publication			
** Review item 4a.** Researchers** (**First authors) admitting adding an honorary author(s) on a publication ** Question 4a.** ** Did you (First author) include an honorary author in your manuscript?	7.1% (157/2,222) (N1)* [95% CI 6.0–8.2] (n = 2222 surveyees) 54.5% (666/1,221) (N3)* [95% CI 51.7–57.3] (n = 1221 surveyees)	N = 1 (McClellan 2019)^32^ N = 1 (Gülen 2020)^30^	16% [95% CI 13–18] (n = 823 respondents)
** Review item 4b.** Researchers (Senior authors) admitting adding an honorary author(s) on a publication ** Question 4b**. ** Did you (Senior author, i.e., last author) include an honorary author in your manuscript?	3.9% (87/2,222) (N1)* [95% CI 3.1–4.8] (n = 2222 surveyees)	N = 1 (McClellan 2019)^32^	11.5% (10/87) [95% CI 5.7–20.1] (n = 87 respondents) ***

*N1: Number of emails with questionnaires sent, N3: Number of questionnaires for which the surveyee was available.

**International Committee of Medical Journal Editors (ICMJE)-based honorary authorship.

***McClellan et al.^32^ assessed review item 4 in both first and senior, i.e., last authors of the same manuscript. Only the answers of the first authors (Q4a) was included in the meta-analysis and the answers of the senior, i.e., last authors (Q4b) were reported separately.

### Results of individual surveys and of syntheses

**Figure 2:**
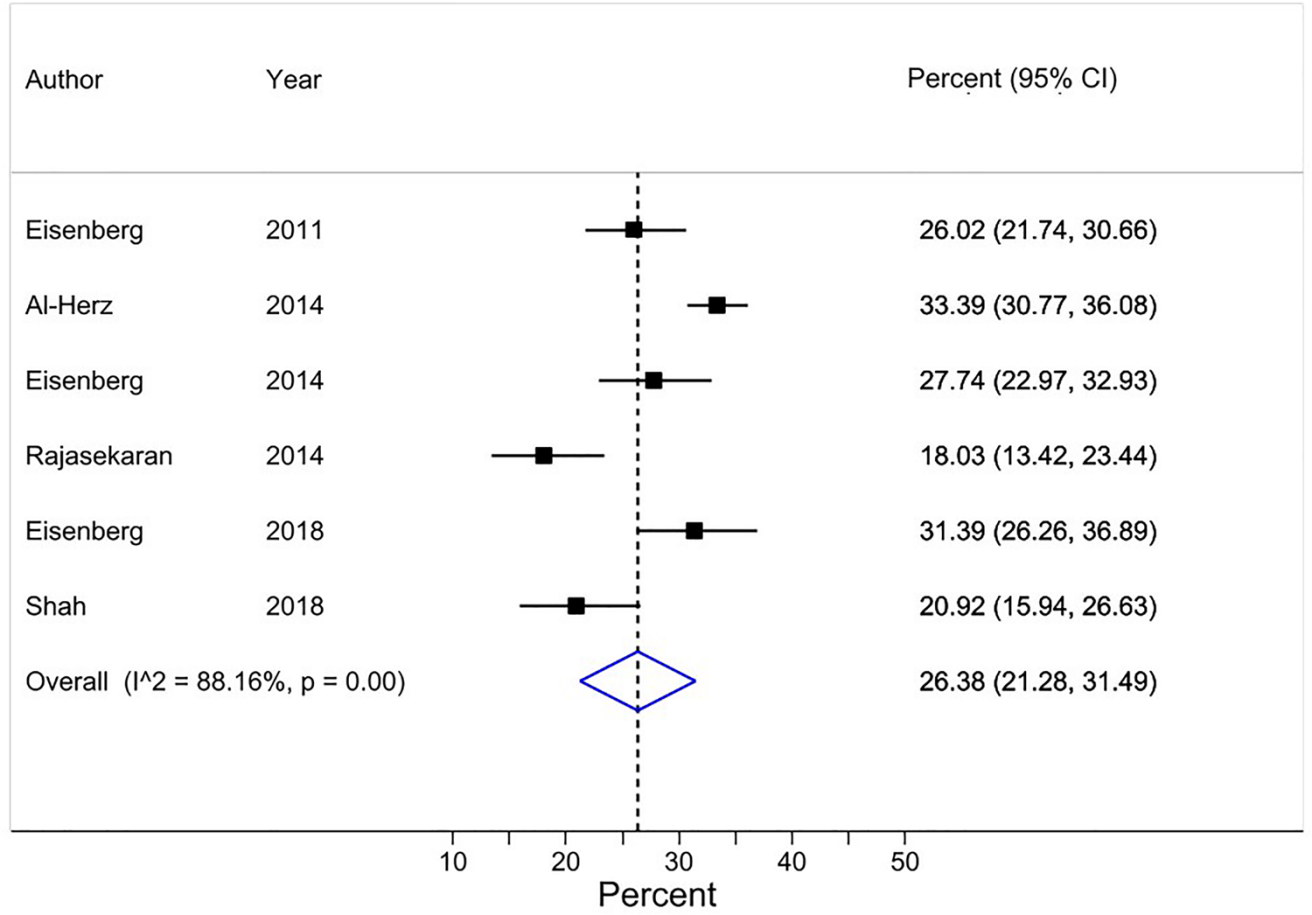
Forest plot for Review item 1a. Perceived honorary authorship of a co-author (without researchers being referred to any specific criteria for authorship).*Effect Size (pooled percentage) 26% [95% CI 21–31]. Heterogeneity χ^2^ = 42.25 [df = 5]; P < 0.001. Variation in pooled percentage attributable to heterogeneity: I^2^ = 88.16%. Between-study variance estimate τ^2^ = 0.00. Test of pooled percentage = 0: z = 10.13; P < 0.001. *The results of each individual survey were based on the answers to the same question regarding a specific publication by the surveyee.

**Figure 3:**
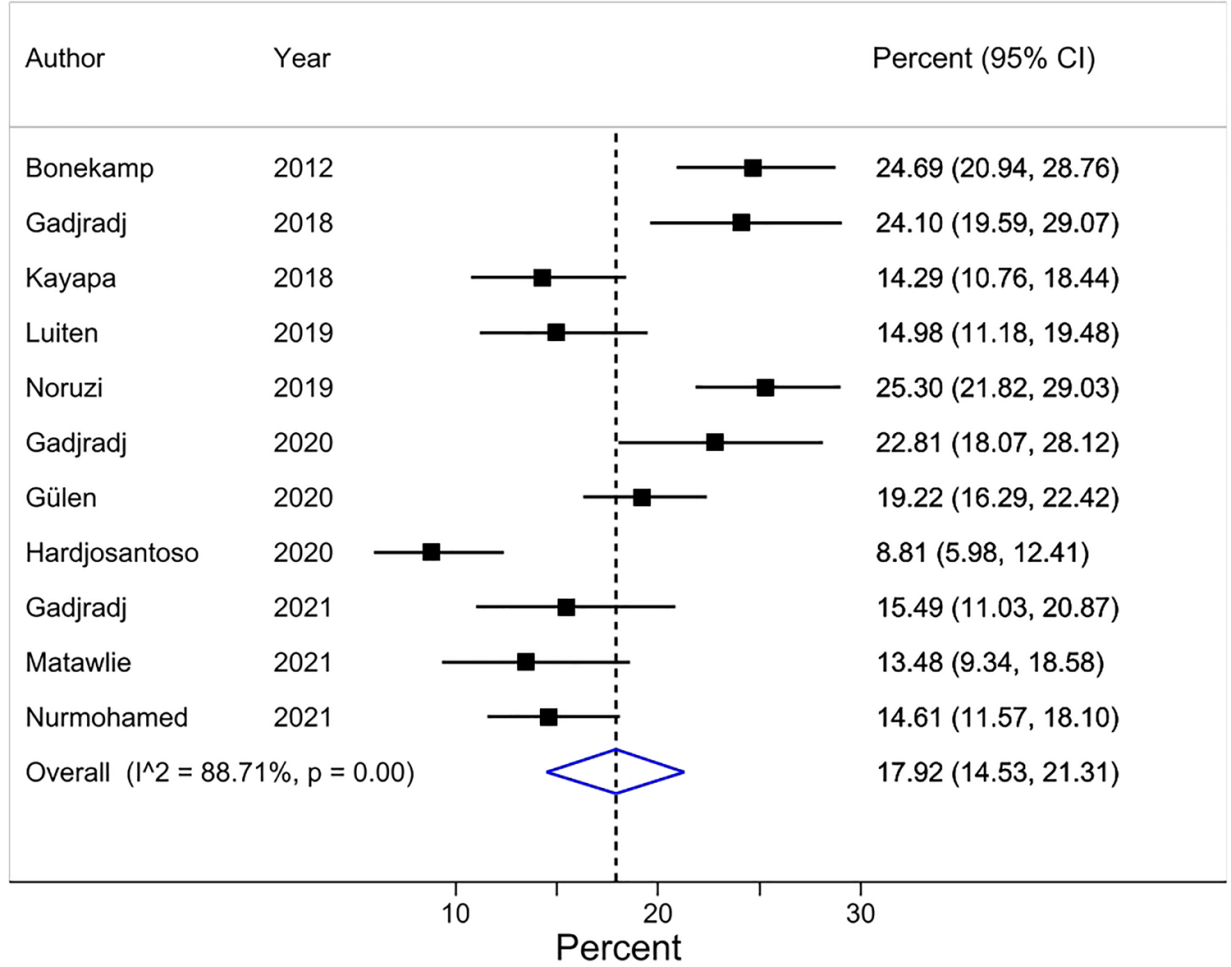
Forest plot for Review item 1b. Perceived honorary authorship based on ICMJE criteria*. Effect Size (pooled percentage) 18.0% [95% CI 15–21]. Heterogeneity χ2 = 85.56 [df = 10]; P < 0.001. Variation in pooled percentage attributable to heterogeneity: I^2^ = 88.71%. Between-study variance estimate τ^2^ = 0.00. Test of pooled percentage = 0: z = 10.37; P < 0.001. *The results of each individual survey were based on the answers to the same question regarding a specific publication by the surveyee.

**Figure 4:**
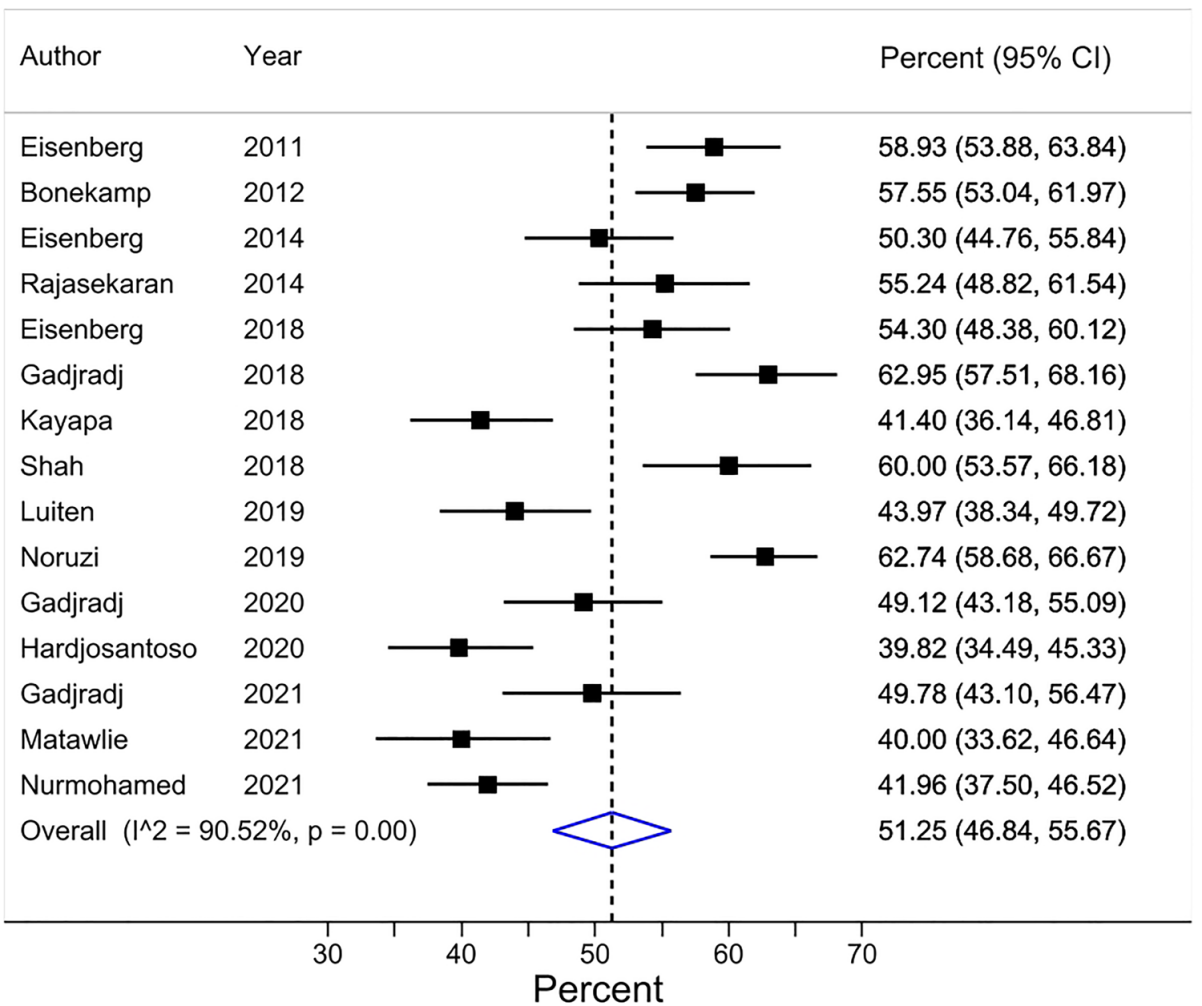
Forest plot for Review item 1c. Honorary authorship based on researchers’ reported contributions of co-authors compared to the ICMJE criteria*. Effect Size (pooled percentage) 51% [95% CI 47–56]. Heterogeneity ^χ2^ = 147.74 [df = 14]; P < 0.001. Variation in pooled percentage attributable to heterogeneity: I^2^ = 90.52%. Between-study variance estimate τ^2^ = 0.01. Test of pooled percentage = 0: z = 22.75; P < 0.001. *The results of each individual survey were based on the answers to the same question regarding a specific publication by the surveyee.

**Figure 5:**
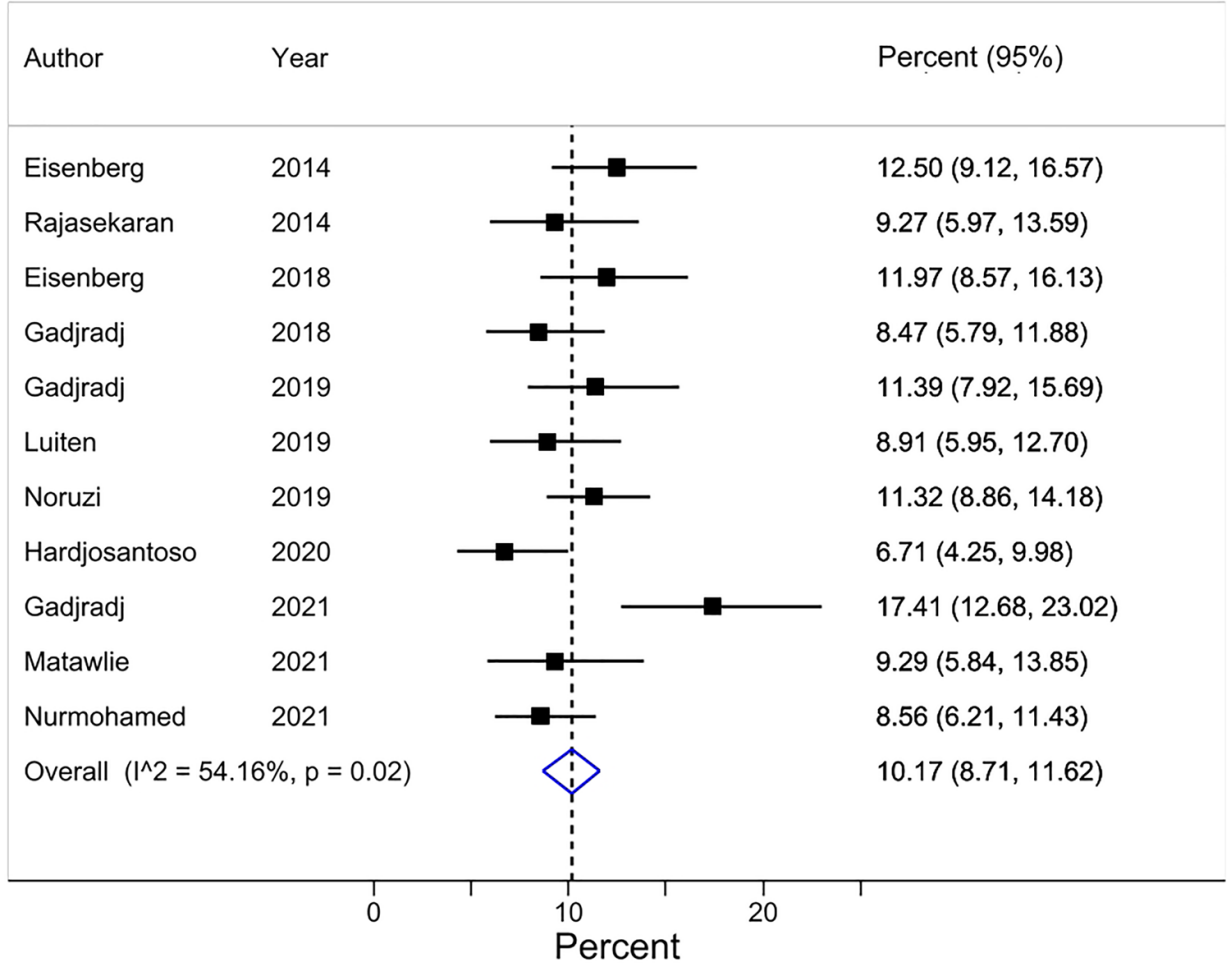
Forest plot for Review item 2. Researchers having been approached by others to include honorary author(s) on a publication*. Effect Size (pooled percentage) 10% [95% CI 9–12]. Heterogeneity χ^2^ = 21.81 [df = 10]; P = 0.02. Variation in pooled percentage attributable to heterogeneity: I^2^ = 54.16%. Between-study variance estimate τ^2^ = 0.00. Test of pooled percentage = 0: z = 13.70; P < 0.001. *The results of each individual survey were based on the answers to the same question regarding a specific publication by the surveyee.

**Figure 6:**
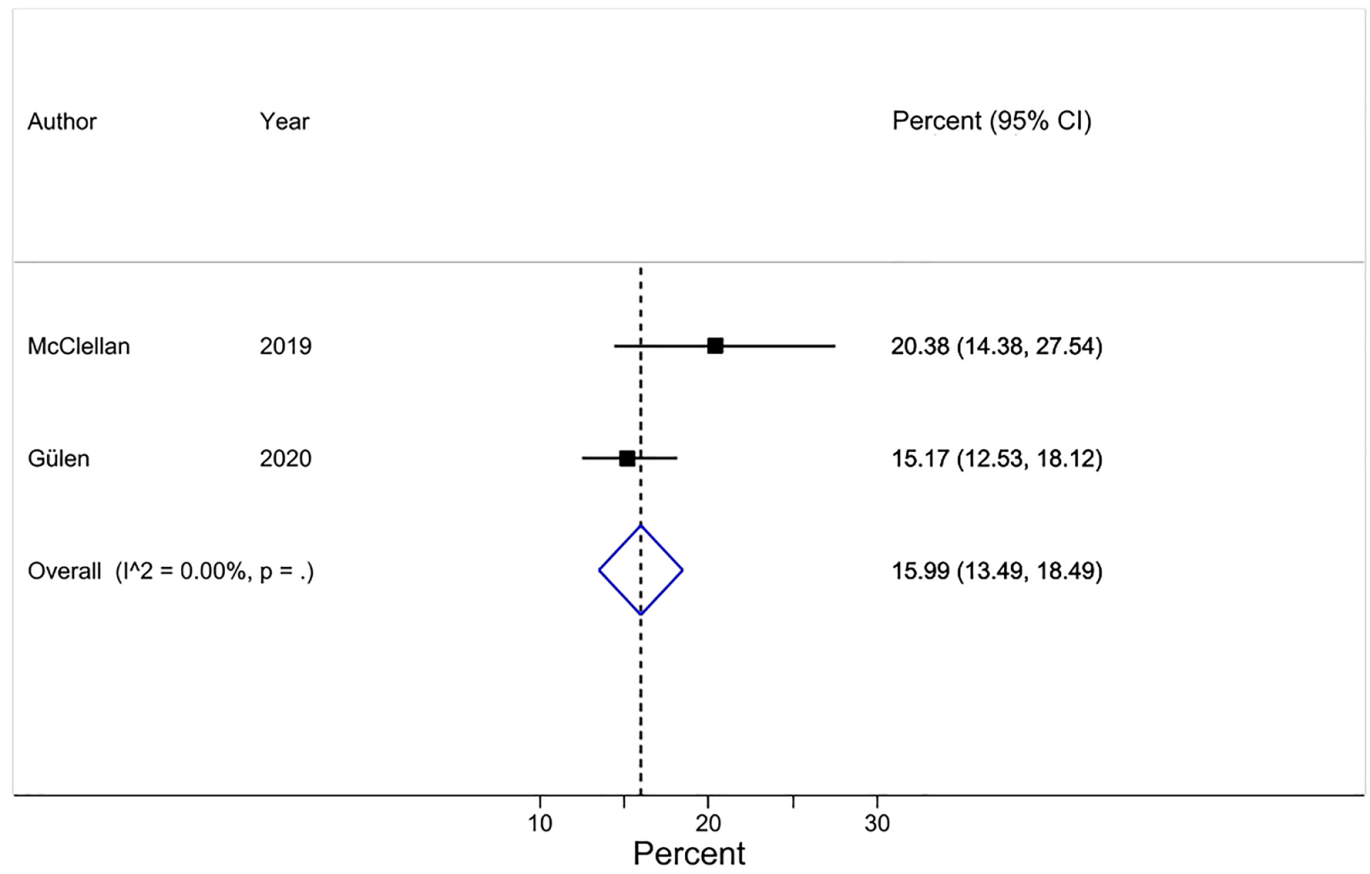
Forest plot for Review item 4a. Researchers (First authors) admitting adding an honorary author(s) on a publication*. Effect Size (pooled percentage) 16% [95% CI 13–18]. Test of pooled percentage = 0: z = 12.53; P < 0.001. *The results of each individual survey were based on the answers to the same question regarding a specific publication by the surveyee.

Surveys used different questions to assess HA. In line with our protocol, we assigned these into the main HA categories in our review. A total of 51 questions provided 51 HA prevalences. Five identical questions (questions 1a, 1b, 1c, 2, and 4a listed in Tables 3 and 4) were used in multiple surveys, which permitted synthesizing of 45 HA prevalences in 5 meta-analyses (using the random effects model). The remaining 6 HA prevalences were obtained from six questions used only once (question 1d, 3a-c, and 4b). Tables 3 and 4 show all questions and results and Figs. 2, 3, 4, 5 and 6 the pertinent forest plots. Additional information on the results is in the Appendix (Additional item L, pages 33–36).

#### Co-author(s) as honorary author(s) on a publication (Review item 1)

##### Perceived HA of a co-author (without researchers being referred to any specific criteria for authorship). (Review item 1a)

A pooled average of 26% [95% CI 21–31] of researchers (based on data from 6 surveys, and a total of 2,758 respondents) perceived that at least one of their co-author(s) on the publication at issue had not contributed sufficiently to deserve authorship (Table 3) (Fig. 2). These judgments were self-perceived and not based on pre-defined criteria for authorship. Three of the 6 surveys were conducted on publications in the field of radiology. In five surveys, first authors were the target population (Appendix Table A17, page 22). The majority of the respondents in these surveys were male (> 51% in the 4 surveys that reported this prevalence) and the percentage of respondents being at least the rank of associate professor or having higher was 32% or more in 4 of the 5 surveys that reported this percentage (Appendix Table A18, page 23).

##### Perceived HA based on ICMJE criteria (Review item 1b)

A pooled average of 18% [95% CI 15–21] of researchers (based on data from 11 surveys, and a total of 4,272 respondents) perceived that at least one (of their) co-author(s) had not contributed sufficiently to deserve authorship on the publication at issue based on the ICMJE criteria for authorship, they were asked to consider (Table 3) (Fig. 3). The 11 surveys on this HA issue were conducted in a broad spectrum of biomedical fields (Appendix Table A17, page 22). Nine of these surveys were conducted on corresponding authors and the majority of respondents were male (> 61% in 8 of the 9 surveys that reported this percentage). The percentage of respondents at least of the rank of associate professors was higher than 32% in the 6 surveys that reported this proportion (Appendix Table A18, page 23).

##### HA based on researchers’ reported contributions of co-authors compared to the ICMJE criteria. (Review item 1c)

A pooled average of 51% (95% CI 47–56, based on 15 surveys and 5111 respondents) of HA was found when researchers were asked to declare their co-author(s’) contributions on the publication at issue (and those were then compared to ICMJE criteria by the researchers conducting the survey) (Table 3) (Fig. 4). These 15 surveys were conducted on researchers from various biomedical disciplines. Nine of these surveys were conducted on corresponding authors, 5 on first authors, and 1 on a mix of corresponding, first, and senior (last) authors. The majority of respondents were male (> 51% in the 11 of 15 surveys that reported this prevalence) and the percentage of respondents ranked at least associate professors was at least higher than 32% in most surveys. Table 3 and the Appendix (Appendix Tables A17 and A18, pages 22 and 23) show additional information.

#### Researchers having been approached by others to include honorary author(s) on a publication (Review item 2)

A pooled average of 10% [95% CI 9–12] of researchers (based on 11 surveys and a total of 3,663 respondents) had been approached by others to include honorary author(s) on the publication at issue (Table 3) (Fig. 5). Surveys asking this question did not report whether they provided researchers with the ICMJE criteria for authorship. The 11 surveys were conducted on a wide spectrum of biomedical publications. Seven of the surveys were conducted on corresponding authors, three on first authors, and 1 on a mix of corresponding, first, and senior (last) authors. The majority of respondents were male and the percentage of respondents that were at least associate professors at least 32% (Table 3, Appendix Table A18, page 23).

#### Researchers admitting being an honorary author(s) on a publication (Review item 3)

We found ranges from 0.15% (1/666) [95% CI 0.0038–0.83]^31^ to 33.3% (67/201) [95% CI 27–40]^32^ of researchers being classified as honorary author(s) based on the type of contribution(s) they reported on the publication at issue (Table 4). These self-declared contributions were made when researchers were told the ICMJE criteria, however, we were unable to meta-analyze these data, because of differently phrased questions, different lists of possible contributions, and even possible overlap of results (Appendix Tables A28–A31, pages 35 and 36).

#### Researchers admitting adding an honorary author(s) on a publication (review item 4)

A pooled average of 16% [95% CI 13–18] of first authors (first authors, based on 2 surveys^31,33^ and a total of 823 respondents) admitted having added an honorary author(s) on the publication at issue (Question 4a) (Table 4) (Fig. 6). In both surveys, first authors declared this when being told the ICMJE criteria for authorship. Additionally, one of these surveys^33^ asked the same question to the last authors on the same manuscript at issue and found that 11.5% (10/87) [95% CI 6–20] of last authors admitted having added an honorary author(s) (Question 4b) (Table 4). We did not pool this result, because the publication at issue was the unit of analysis and we therefore could not use the results for both first authors and last authors in the same meta-analysis (Appendix Tables A26 and A32, pages 34 and 36).

#### Researchers admitting having approached others to include honorary author(s) on a publication (review item 5)

No surveys assessed this outcome.

### Investigation of heterogeneity and sensitivity analyses

Visual inspection of the forest plots, the tests of homogeneity (Chi-squared), and the estimate of between study variance (tau^2^) of the 5 quantitatively synthesized results all demonstrated evidence of between-study heterogeneity (Figs.  2, 3, 4, 5 and 6). The variation in the prevalence proportions attributable to heterogeneity (I^2^) was larger than 88% for 3 out of 5 main outcomes (Figs.  2, 3, 4, 5 and 6). Meta-regression showed that the prevalence of HA based on researchers’ reported contributions of co-authors compared to the ICMJE criteria decreased by 1.3% per year between 2011 and 2021 [95% CI − 2.5 to − 0.07], p = 0.04) (Question 1c). The percentage of respondents being at least associate professors had no association with the prevalence of researchers that perceived other co-author(s) as honorary author(s) on a publication based on a list of co-author’s contributions (review question 1c) (Appendix Table A34, page 40).

Three of 11 subgroup analyses found a significant (p < 0.05) association between an explanatory variable and the quantitatively synthesized results for a specific survey question. When the survey was conducted by a research group whose first author was affiliated with a research institute in the Netherlands it was less likely (Q (1) = 5.60, p = 0.018) that researchers perceived other co-author(s) as honorary author(s) on a publication based on a list of co-author’s contributions (review question 1c) than when these surveys were conducted in countries other than the Netherlands (Table Question 1c). When the survey was conducted within 1 year after the publication of the manuscript, perceived honorary authorship based on ICMJE criteria (Question 1b) and honorary authorship based on researchers’ reported contributions of co-authors compared to the ICMJE criteria (Question 1c) were less likely (respectively Q (1) = 6.62, p = 0.01 for question 1b and (Q (1) = 5.79, p = 0.016 for question 1c) than when these surveys were conducted after more than 1 year (Table Question 1b and 1c). The Appendix (Additional item M, pages 37–43) reports all pre-specified explanatory variables that were assessed, why some of the predictors were excluded and has details on all investigations of heterogeneity. Sensitivity analyses were not conducted, because the sources in which eligible surveys were identified and the survey design and quality of the included surveys were similar.

### Non-reporting biases in syntheses

An overall judgement about risk of bias due to missing results in a synthesis (non-reporting biases)^26^ was ‘moderate’ probability of risk of bias for 9 review items and ‘high’ probability of risk of bias for 2 review items. The Appendix (Additional item N, pages 44–47) reports the methods and results and explanations for the overall judgment on non-reporting biases.

### Certainty of evidence

The certainty of evidence (GRADE)^28^ were either low or very low for all outcomes of this systematic review (Table 5). The rationales for assigning certainty grades are in the Appendix (Additional item O, page 49).

**Table 5:** Summary of findings.

Review items	Prevalence	# of respondents and surveys	Certainty of the evidence (GRADE)*
Surveyee: Any author on the author list of a scientific publication, e.g., first, last, corresponding author, that was invited to participate in a survey on at least one of our review items. Settings: Any			
**Review item 1a (Question 1a).** Perceived honorary authorship of a co-author (without researchers being referred to any specific criteria for authorship)	26% [95% CI 21–31]	2,758 respondents 6 surveys	⊕ ⊝ ⊝ ⊝ **Very low** Due to risk of bias, inconsistency, imprecision, and moderate risk of non-reporting biases
**Review item 1b (Question 1b).** Perceived honorary authorship based on ICMJE criteria	18% [95% CI 15–21]	4,272 respondents 11 surveys	⊕ ⊝ ⊝ ⊝ **Very low** Due to risk of bias, inconsistency, imprecision, and moderate risk of non-reporting biases
Review item 1c (Question 1c). ** Honorary authorship based on researchers’ reported contributions of co-authors compared to the ICMJE criteria	51% [95% CI, 47–56]	5,111 respondents 15 surveys	⊕ ⊝ ⊝ ⊝ **Very low** Due to risk of bias, inconsistency, imprecision, and high risk of non-reporting biases
**Review item 1c (Question 1d).** ** Honorary authorship based on researchers’ reported contributions of co-authors compared to the ICMJE criteria	22.2% (148/666) [95% CI, 19–26]	666 respondents 1 survey (Gülen 2020)^30^	⊕ ⊕ ⊝ ⊝ **Low** Due to risk of bias, imprecision, and moderate risk of non-reporting biases
**Review item 2 (Question 2).** ***Researchers having been approached by others to include honorary author(s) on a publication	10% [95% CI, 9–12]	3,663 respondents 11 surveys	⊕ ⊝ ⊝ ⊝ **Very low** Due to risk of bias, inconsistency, and high risk of non-reporting biases
**Review item 3a (Question 3a).** ** Researchers admitting being an honorary author(s) on a publication based on a list of author’s contributions. Not contributed: First author did not conceive or design the work, conduct literature search, or analyze and interpret data	0.15% (1/666) (95% CI 0.0038–0.83]	666 respondents 1 survey (Gülen 2020)^30^	⊕ ⊕ ⊝ ⊝ **Low** Due to risk of bias, imprecision, and moderate risk of non-reporting biases
**Review item 3b (Question 3b)**. ** Researchers admitting being an honorary author(s) on a publication based on a list of author’s contributions. Not contributed: First author did not draft or revise the review	0.15% (1/666) [95% CI 0.0038–0.83]	666 respondents 1 survey (Gülen 2020)^30^	⊕ ⊕ ⊝ ⊝ **Low** Due to risk of bias, imprecision, and moderate risk of non-reporting biases
**Review item 3c (Question 3c).** ** Researchers admitting being an honorary author(s) on a publication based on a list of author’s contributions. Not contributed: First author did not give the final approval	7.2% (48/666) (95% CI 5.4–9.4]	666 respondents 1 survey (Gülen 2020)^30^	⊕ ⊕ ⊝ ⊝ **Low** Due to risk of bias, imprecision, and moderate risk of non-reporting biases
Review item 3d (Question 3d). ** Researchers admitting being an honorary author(s) on a publication based on a list of author’s contributions	33.3% (67/201) [95% CI 26.9–40.3]	201 respondents 1 survey (Ilakovac 2007)^31^	⊕ ⊝ ⊝ **Very low** Due to risk of bias, imprecision, and moderate risk of non-reporting biases
Review item 4 (Question 4a). ** Researchers (first authors) admitting adding an honorary author(s) on a publication	16% [95% CI 13–18]	823 respondents 2 surveys	⊕ ⊕ ⊝ ⊝ ⊝ **low** Due to risk of bias, inconsistency, and moderate risk of non-reporting biases
Review item 4 (Question 4b). ** Researchers (Senior, i.e., last authors) admitting adding an honorary author(s) on a publication	11.5% (10/87) [95% CI, 5.7–20.1]	87 respondents (McClellan 2019)^32^	⊕ ⊝ ⊝ ⊝ **Very low** Due to risk of bias, imprecision, and moderate risk of non-reporting biases

Prevalence of honorary authorship issues in a publication on which the survyee was surveyed.

*The rationales for the certainty grades (GRADE) are given in the Appendix.

**International Committee of Medical Journal Editors (ICMJE)-based honorary authorship.

***Not specified whether it was perceived or ICMJE-based honorary authorship or both.

## Discussion

We identified 19 surveys assessing honorary authorship in health sciences using 51 different operationalizations and statistically pooled 45 of those into 5 review outcomes. Results indicate that HA prevalence was 26% when respondents were asked if there are honorary authors on their publication at issue, and not explicitly informing authors about criteria for authorship. The pooled prevalence was 18% when they were asked the same question but ICMJE criteria were disclosed to them, and 51% when respondents were asked to declare their co-author(s) contributions and these contributions were then compared to the ICMJE criteria independently. This indicates that how questions are asked may affect HA estimates, but also that what researchers perceive as HA and may differ from how authorship is defined using ICMJE criteria. Previous qualitative research also revealed these effects^34^.

Nevertheless, the apparent high prevalences of HA (regardless of how questions are phrased and definitions used), confirm previous findings that authorship issues are among the most prevalent Questionable Research Practices (QRPs) in science, affecting both young and old researchers^35,36^. A slight ray of hope is that we also found an indication that the prevalence of HA when respondents are asked to declare co-author(s) contributions and these are compared to the ICMJE criteria has been decreasing over time. While our study was not designed to detect reasons for this phenomenon, we hope it is due to the increase in raising awareness of QRPs, and greater emphasis on research integrity integration, as well as promotion of authorship templates at the start of a research project^37^. We also found that 10% of researchers stated they had been approached by others to include honorary author(s) on the paper at issue, and 16% of researchers who admitted having added an honorary author to that paper. These pooled prevalences are lower than those mentioned above, which is not surprising, as meta-analyses of falsified, fabricated or plagiarized data have also shown that researchers perceive others to be more fallible to committing the same fallacies than they themselves^38,39^.

Previous reviews on HA were narrative^1,40,41^, integrative^42^ or also systematic^36^, but reviewed predominantly other issues than those assessed in our systematic review. During our study selection, multiple surveys were excluded, because they had different objectives such as estimating the occurrence of ‘HA issues in past publications’^43–47^. Compared to that work, obviously, our focus on ‘the prevalence of HA issues in the publication on which researchers were surveyed’, yielded lower estimates.

The limitations of our study can be looked at on 2 levels, one for the review itself, and the second regarding the surveys we pooled. Regarding the review itself, we have assessed results of surveys only in the health sciences, and further research should be conducted on other disciplines. In addition, as our search strategy included full words such as authorship as did Marušić’ systematic review^36^, we may have missed studies that did not include those words in their abstract or title. As for the surveys, we undertook pooling of results even though our findings indicated the low methodological quality of surveys. We did so intentionally, as surveys remain the main mechanisms for estimating the prevalence’s of misconduct and QRP, and the low response rates and inadequate reporting has been found for surveys addressing many topics, which also led to creation of several survey reporting guidelines. When using the pooled estimates, we therefore recommend always keeping in mind that they originate from survey research and its inherent deficiencies^48^.

HA inflates the publication output by honorary authors, which could benefit their career and even income. HA is not a victimless crime, because it dilutes the output of those authors that did the work. It also harms the trust between researchers, creating stressful relationships and poor collaboration, which could even slow down the research progress. It is very likely that it also affects the research culture, which a recent a recent survey has found to be lacking^49^. Measuring the prevalence of HA issues is important, because it demonstrates the need to implement strategies to address these problems and shows whether these strategies are effective. The high prevalence of various honorary authorship issues identified in this systematic review of survey research showed the need tackle these problems. Multiple strategies to reduce HA issues such as courses in research integrity, publication ethics, authorship responsibilities of the pertinent stakeholders^1,6^, authorship/contribution discussions during the various research phases^7^, adopting and fine-tuning of authorship contributor criteria^7,50^, sanctions for honorary authorship, and considering and implementing new principles for assessing the quality of scientists^51^ have been proposed, but call for testing. This systematic review also demonstrated the need to improve the quality of survey research on HA issues.

## Conclusions

The pooled estimates in this systematic review appear to confirm the idea that honorary authorship is a highly prevalent questionable research practice in the health sciences. However, due the poor quality of the surveys yielding the data, this conclusion cannot be made with certainty. Future research should be aimed at testing and implementing methods aimed at reducing this practice, and cultivating a culture that values quality over the quantity of publications.

### Supplementary Information

Supplementary Information.

## Data Availability

All raw and analyzed data of this systematic review are reported in the manuscript and Appendix or were deposited in OSF Storage https://osf.io/692rb. We will respond rapidly to requests for additional clarifications on our data. Requests can be made to the corresponding author (RMR) at reyndersmail@gmail.com. Protocol registration and publication: The protocol was registered in Open Science Framework. Link: https://osf.io/5nvar/. Reference of the published protocol: Meursinge Reynders R, Ter Riet G, Di Girolamo N, Malički M. Honorary authorship in health sciences: a protocol for a systematic review of survey research. Syst Rev. 2022 Apr 4;11(1):57. 10.1186/s13643-022-01928-1. PMID: 35379330; PMCID: PMC8978359. Link to the published protocol: https://systematicreviewsjournal.biomedcentral.com/articles/10.1186/s13643-022–01928-1.

## Abbreviations

HA: Honorary authorship
ICMJE: International Committee of Medical Journal Editors
OSF: Open science framework
QRP: Questionable research practice

## Code availability

This manuscript or parts of it have not been submitted concurrently to any other journal or in any other form or language. This manuscript or parts of it have not been peer reviewed by any other journal previously. The lead author (RMR) affirms that the manuscript is an honest, accurate, and transparent account of the study being reported; that no important aspects of the study have been omitted; and that any discrepancies from the study as originally planned in our published protocol have been explained. The appendix are all web-only material. There was no patient and public involvement. The authors did not use generative AI and AI assisted technologies in the writing process.
