# Supplementary material for: Honorary authorship is highly prevalent in health sciences: systematic review and meta-analysis of surveys
Source: Sci Rep. 2024 Feb 22;14:4385. doi: 10.1038/s41598-024-54909-w (PMC10883936; doi:10.1038/s41598-024-54909-w)
Supplement: Supplementary file 1 — Supplementary Information. [file 41598_2024_54909_MOESM1_ESM.pdf]

Appendix

This is an appendix to ***'Honorary authorship is highly prevalent in health sciences: systematic review and meta-analysis of surveys'*** *by* Reint Meursinge Reynders, Gerben ter Riet, Nicola Di Girolamo, Davide Cavagnetto, Mario Malički. This appendix reports additional items for both the methods and for the results section.

Contents of additional items for the methods section

**Additional item**	**Description**	**Page(s)**
Additional item A	Differences between the protocol and the final systematic review	2
Additional item B	Eligibility criteria	4
Additional item C	Search strategies for PubMed, Lens.org, and Dimensions.ai	5
Additional item D	Data collection forms	6-10
Additional item E	Quality assessment: Quality checklist for surveys on Honorary Authorship (HA) items, Guidance for completing the quality checklist for surveys on HA issues, Guidance for rating the overall confidence in the results of a survey	11-14
Additional item F	Occurrence measures and synthesis methods: Defining outcomes, Criteria for a quantitative synthesis, Investigation of heterogeneity	15-16
Additional item G	(Non) reporting bias assessment	17
Additional item H	Guidance for grading the certainty or quality of evidence for a review item	18-19

Contents of additional items for the results section

**Additional item**	**Description**	**Page(s)**
Additional item I	Included surveys and characteristics of included surveys	20-24
Additional item J	Excluded studies with rationale	25-28
Additional item K	Rating of the overall confidence in the results of a survey	29-32
Additional item L	Results of the survey questions	33-36
Additional item M	Investigation of heterogeneity	37-43
Additional item N	Non-reporting biases	44-47
Additional item O	Certainty of the evidence	48-49
Additional item P	References for the Appendix	50-53
Additional item Q	Legends for the tables of the Appendix	54-55

Methods

Additional item A. Differences between the protocol and the final systematic review

The following changes to the protocol were implemented:

- For the study selection, data extraction, and quality assessments, Davide Cavagnetto replaced Nicola Di Girolamo, because both Reint Meursinge Reynders and Davide Cavagnetto collaborate daily at the same institutions. Davide Cavagnetto was calibrated through pilot tests for all three procedures. For the pilot of study selection we used a sample of records to screen. For the pilot of the data extraction and quality assessment we used a sample of surveys to be reviewed.
- We conducted meta-analysis although one or more criteria precluding such analyses were sometimes met. For example, we undertook meta-analyses even when the quality of the results of one or more of the included outcomes was rated as 'critically low' or when high heterogeneity and inconsistency (I^2>50%) were present [1]. We made this decision, because high heterogeneity and low quality is common in survey research. Further, while underway in the review process we believed that the pooled estimates probably reflect a lower boundary of HA (believing that underreporting is the most likely mechanism of bias). If that were true, pooled estimates are useful to convey the seriousness of the problem in research publication ethics. We warned readers of this systematic review to beware of basing firm conclusions on the summary estimates of these meta-analyses in light of the seemingly low-quality data.
- We omitted double arcsine transformation prior to statistical pooling, because recent publications on this topic indicate that double arcsine transformation may invalidate meta-analyses of proportions [2,3].
- We conducted subgroup analyses for two additional explanatory variables to assess (1) whether risk of multiple submissions of questionnaires by surveyees or (2) whether ≥ 50% of the

surveyees being male were predictors for the results for questions 1b, 1c, and 2 (Additional item M).

- We could not build generalized linear mixed models to assess which factors contribute by how much to each of the outcomes reviewed, because individual respondent data were not reported in any of the 19 included surveys.

Additional Item B. Eligibility criteria

Appendix Table A1 presents the 'Inclusion and exclusion criteria' the eligibility criteria for domain, study designs, participants, survey instruments, outcomes, time points, setting, language, publication status, and publication dates [4,5].

Appendix Table A1. Inclusion and exclusion criteria

Item	Inclusion criteria	Exclusion criteria
**Domain**	• Health sciences as defined in Table 1.	
**Study designs**	• Studies including at least one survey according to its definition in Table 1.	• Surveys in which it was unclear what questions were used to assess review items 1-5, i.e., surveys which did not report or whose authors were unreachable or did not provide exact questions asked in the survey
**Participants**	• Any author on the author list of a scientific publication, e.g., first, last, corresponding author etc., that was invited to participate in a survey on at least one of our authorship items.	
**Survey instruments**	• Surveys based on questionnaires for self-completion.<br>• Surveys administered by email, internet platforms, by post, or by hand.<br>• We will only consider closed surveys, i.e., surveys open to a specific sample of participants selected by the investigators.<br>• Surveys with or without incentives to complete it.	• Focus groups discussions and one-to one interviews.
**Outcomes**	• One or more of the outcomes on authorship issues listed in our objectives for review items 1-5 (Table 1).<br>• Both self-and non-self-reported outcomes on authorship issues.	• Outcomes that were not reported as prevalence statistics or were not given in a format that such statistics could be calculated.
**Time point**	• Any time point for measuring outcomes will be eligible, i.e., we will not set exclusion criteria whether an article on which the surveyee was questioned was published 1, 2, 3 etc. years previously.	
**Setting**	• Any	
**Language**	• Any	
**Publication status**	• Peer-and non-peer-reviewed manuscripts.	
**Publication dates**	• Articles published from bibliography inception onwards.	

Additional item C. Search strategies for PubMed, Lens.org, and Dimensions.ai

Appendix Table A2. Search strategies for PubMed, Lens.org, and Dimensions.ai

PubMed

Date search: January 5 2023
Results: 356

Search strategy: (contributorship[Title/Abstract] OR authorship[Title/Abstract]) AND (survey[Title/Abstract] OR questionnaire[Title/Abstract])

Link:https://pubmed.ncbi.nlm.nih.gov/?term=%28contributorship%5BTitle%2FAbstract%5D+OR+authorship%5BTitle%2FAbstract%5D%29+AND+%28survey%5BTitle%2FAbstract%5D+OR+questionnaire%5BTitle%2FAbstract%5D%29

Lens.org

Date search: January 5 2023
Results: 498

Search strategy: (title:(authorship OR contributorship) OR abstract:(authorship OR contributorship)) AND (title:(survey OR questionnaire) OR abstract:(survey OR questionnaire))

Filters: Field of Study = (Medicine, Medical education, Family medicine, Alternative medicine, Nursing, Nurse education)

Link:
https://www.lens.org/lens/search/scholar/list?q=(title:(authorship%20OR%20contributorship)%20OR%20abstract:(authorship%20OR%20contributorship))%20AND%20(title:(survey%20OR%20questionnaire)%20OR%20abstract:(survey%20OR%20questionnaire))&p=0&n=10&s=_score&d=%2B&f=false&e=false&l=en&authorField=author&dateFilterField=publishedYear&orderBy=%2B_score&presentation=false&stemmed=true&useAuthorId=false&fieldOfStudy.must=Medicine&fieldOfStudy.must=Medical%20education&fieldOfStudy.must=Family%20medicine&fieldOfStudy.must=Alternative%20medicine&fieldOfStudy.must=Nursing&fieldOfStudy.must=Nurse%20education

Dimensions.ai

Date search: January 5 2023
Results: 1098

Search strategy: (contributorship or authorship) and (survey or questionnaire)

Filter: Fields of Research: Medical and Health Sciences

Link:
https://app.dimensions.ai/discover/publication?search_mode=content&search_text=(contributorship%20or%20authorship)%20and%20(survey%20or%20questionnaire)&search_type=kws&search_field=full_search

Additional item D Data collection forms

All entries of the data collection forms were collected in Excel spreadsheets.

Appendix Table A3. Data collection forms at the study level in surveys on HA issues*

Entry	Description
Year	Report the year of the publication of the article.
Reference	Report the full reference.
Journal	Report the name of the pertinent journal.
IF (year)	Report the impact factor of the journal. We will report the latest impact factor and the year in which this impact factor was assigned.
Language	Report the language of the article.
Number of authors	Report the number of authors in the article.
Affiliation	Report the type of the first affiliation, i.e., university, industry, care facility, other.
Country first affiliation and name of the first affiliation	Report the country of the first affiliation listed and the name of the institute.
Conflict-of-interest statement reported	Was a conflict-of-interest statement reported? Answer: Yes/No/Unclear. If No, give the rationale.
No potential conflict of interest and funding issues	Were there no potential conflict of interests or funding issues that could have affected the outcomes of the survey? Answer: Yes/No/Unclear. If No, give the rationale.
Registration/publication of the protocol	Was the protocol registered or published a priori? Answer: Yes/No/Unclear. If Yes, we will also extract where and when the protocol was registered.
Institutional Review Board (IRB) approval	Report whether the survey was approved by an IRB. Answer: Yes/No/Unclear.
Institutional Review Board (IRB) exemption	Report whether the survey received an exemption from IRB. Answer: Yes/No/Unclear.
Limitations reported	Did the authors report the limitations of the survey? Answer: Yes/No/Unclear.
To do or contact authors	Report what actions should be undertaken to obtain additional information and whether it is necessary to contact the authors and if so report what information should be obtained.

*We assigned 'Unclear' when too few details were reported in the manuscript or additional files to make a judgment of assigning 'Yes' or 'No'.

Appendix Table A4. Data collection forms at the eligibility level in surveys on HA issues*

Target population	Report the type of target population of the survey, e.g., first or corresponding author or any other author. The target population further refers to characteristics such as assistant, associate, or full professor or head of a department etc.
Target field and context	Report the target field and context of the survey. For the field we refer to e.g., neurology or plastic surgery. For the context we refer to articles, journals, and publication dates on which the target population was surveyed.

*We assigned 'Unclear' when too few details were reported in the manuscript or additional files to make a judgment of assigning 'Yes' or 'No'.

Appendix Table A5. Data collection forms at the sampling level in surveys on HA issues*

Sampling technique	Report what sampling technique was used, e.g., consecutive, i.e., consecutive subjects were sampled, random, non-random, open link on a website, cluster (multistage), sampling weights, strata etc.

*We assigned 'Unclear' when too few details were reported in the manuscript or additional files to make a judgment of assigning 'Yes' or 'No'.

Appendix Table A6. Data collection forms at the survey methods level in surveys on HA issues*

Survey delivery (Subitem 1)	Report the methods of survey delivery, e.g., email, post, telephone etc.
Incentives (Subitem 2)	Report whether incentives were given to surveyees to complete the questionnaires. Answer: Yes/No/Unclear. If Yes, give the type of incentives.
Timeframe (Subitem 3)	Report whether the timeframe between the year of publication of a research publication and the date of the survey on this publication was reported. Answer: Yes/No/Unclear. If Yes, give the time frame.
Multiple (desired) submissions of surveys by the same surveyee (Subitem 4)	Report whether the manuscript reported that the surveyee completed more than 1 survey, e.g., when surveyees who have published multiple articles in the eligible time span were asked to submit a questionnaire for each published article. Answer: Yes/No/Unclear. If Yes, report on these multiple submissions.
Methods to prevent multiple (undesired) submissions of surveys by the same surveyee (Subitem 5)	Report whether methods were implemented for preventing the submitting of more than one questionnaire by the same surveyee when he/she was invited to submit only one. Answer: Yes/No/Unclear. If yes, give the methods.

*We assigned 'Unclear' when too few details were reported in the manuscript or additional files to make a judgment of assigning 'Yes' or 'No'.

Appendix Table A7. Data collection forms at the surveyee level in surveys on HA issues*

Characteristics of the responding surveyees	Report distribution characteristics of responding surveyees such as: Sociodemographic characteristics: distributions of age (measure of central tendency (mean, p50), measure of dispersion [SD, IQR]), Sex/Gender**, career level such as PhD students, seniority etc. country, number of published papers, additional characteristics.
Characteristics of the non-responding surveyees	Were the characteristics of the non-responding surveyees defined? Answer: Yes/No/Unclear.

*We assigned 'Unclear' when too few details were reported in the manuscript or additional files to make a judgment of assigning 'Yes' or 'No'.

**We reported how the terms sex/gender were used and considered (with complete gender/sex breakdown for all considered categories) in the design of each included survey [6]

Appendix Table A8. Data collection forms at the response rate level in surveys on HA issues*

Number of emails with questionnaires on HA issues sent (N1)	The total number of emails with questionnaires on HA issues sent.
Number of emails with questionnaires on HA issues not bounced (N2)	The total number of emails with questionnaires on HA issues sent that had surveyees with valid email addresses.
Number of questionnaires for which the surveyee was available (N3)	The total number of emails with questionnaires sent to assess HA issues with surveyees with valid email addresses and for which the surveyee was available. Unavailability can be the result of, e.g., automated responses such as 'out of office', 'study leave', 'on strike', 'vacation leave', 'maternity leave' etc.
Number of partly or completely answered questionnaires (N4)	The total number of questionnaires on HA issues received back in which the questions were answered (either partial or completely).
Number of completely answered questionnaires (N5)	The total number of questionnaires on HA issues received back in which all questions were answered.
Overall response rates in questionnaires on HA issues	N4 or N5/N1, N2, or N3
Sex/Gender prevalence among the respondents*	For example: Number of males/N4 or N5, e.g., the number of males/N4 or N5 or the number of females/N4 or N5.

*We reported how the terms sex/gender were used and considered (with complete gender/sex breakdown for all considered categories) in the design of each included survey [6]

Appendix Table A9. Data collection forms for review item 1*

Review item 1 defined	Was review item 1 defined? Answer: Yes/No/Unclear.
Definition review item 1	Report the definition of review item 1.
Reporting of survey question to assess review item 1	Did the survey (or any additional file) report the survey question to assess review item 1? Answer: Yes/No/Unclear.
Survey question to assess review item 1	Report the survey question to assess review item 1.
Type of answering scale	Yes/No/Unclear, Likert type answers etc.
Validation of the survey question to assess review item 1	Was the question to assess review item 1 validated (tested) a priori e.g., through pilot testing or used in previous surveys? Answer: Yes/No/Unclear.
Number of questionnaires that answered the question on review item 1 (N6)	The total number of questionnaires received back in which the question on review item 1 was answered.
Reporting of the response rate on review item 1	Was the initial sample size and the number of questionnaires that answered the question on review item 1 reported? Answer: Yes/No/Unclear. Initial sample size refers to any initial sample size, i.e., N1, N2, N3, N4, or N5
Response rate on review item 1	N6/N1, N2, N3, N4 or N5
Magnitude of the response rate on review item 1	Was the response rate on review item 1 higher than 50% [7]? Answer: Yes/No/Unclear.
Number of questionnaires in which the respondents reported review item 1 (N7)	The number of questionnaires in which the respondents reported review item 1, i.e., perceiving other co-author(s) as honorary author(s) on a publication.
Prevalence of review item 1 (**Primary outcome**)	N7/N6
Sample size on the prevalence of review item 1	Was the sample size adequate for the prevalence statistic of review item 1? Answer: Yes/No.
Approach to statistical analysis for review item 1	Report the approach to statistical analysis (regression, group comparisons) for review item 1.
Complete reporting of outcome measures on review item 1	Were the complete outcome measures given for review item 1, i.e., were the numerators and denominators reported? Answer: Yes/No.
Weighting of the survey results for review item 1	Were the results of the survey for review item 1 weighted, i.e., corrected for selective nonresponse? Answer: Yes/No/Unclear. If Yes, describe the methods.
Additional issues	Report whether additional issues on review item 1 could have affected outcomes. Answer: Yes/No/Unclear. If Yes, explain.

*We assigned 'Unclear' when too few details were reported in the manuscript or additional files to make a judgment of assigning 'Yes' or 'No'.

Appendix Table A 10. Data collection forms for review item 2*

Review item 2 defined	Was review item 2 defined? Answer: Yes/No/Unclear.
Definition review item 2	Report the definition of review item 2
Reporting of survey question to assess review item 2	Did the survey (or any additional file) report the survey question to assess review item 2? Answer: Yes/No/Unclear.
Survey question to assess review item 2	Report the survey question to assess review item 2.
Type of answering scale	Yes/No/Unclear, Likert type answers etc.
Validation of the survey question to assess review item 2	Was the question to assess review item 2 validated (tested) a priori e.g., through pilot testing or used in previous surveys? Answer: Yes/No/Unclear.
Number of questionnaires that answered the question on review item 2 (N8)	The total number of questionnaires received back in which the question on review item 2 was answered.
Reporting of the response rate on review item 2	Was the initial sample size and the number of questionnaires that answered the question on review item 2 reported? Answer: Yes/No/Unclear. Initial sample size refers to any initial sample size, i.e., N1, N2, N3, N4, or N5
Response rate on review item 2	N8/N1, N2, N3, N4 or N5
Magnitude of the response rate on review item 2	Was the response rate on review item 2 higher than 50% [7]? Answer: Yes/No/Unclear.
Number of questionnaires in which the respondents reported review item 2 (N9)	The number of questionnaires in which the respondents reported review item 2, i.e., perceiving other co-author(s) as honorary author(s) on a publication.
Prevalence of review item 2* (**Primary outcome**)	N9/N8
Sample size on the prevalence of review item 2	Was the sample size adequate for the prevalence statistic of review item 2? Answer: Yes/No.
Approach to statistical analysis for review item 2	Report the approach to statistical analysis (regression, group comparisons) for review item 2
Complete reporting of outcome measures on review item 2	Were the complete outcome measures given for review item 2, i.e., were the numerators and denominators reported? Answer: Yes/No.
Weighting of the survey results for review item 2	Were the results of the survey for review item 2 weighted, i.e., corrected for selective nonresponse? Answer: Yes/No/Unclear. If Yes, describe the methods.
Additional issues	Report whether additional issues on review item 2 could have affected outcomes. Answer: Yes/No/Unclear. If Yes, explain.

*We assigned 'Unclear' when too few details were reported in the manuscript or additional files to make a judgment of assigning 'Yes' or 'No'.

Note: Data extraction forms to extract review items 3-5 were based on the same format as those reported in Appendix Table A9 and Appendix Table A10.

Additional item E Quality assessment

Quality checklist for surveys on HA items

The 14-item quality checklist presented below was developed and tailored to our research questions on HA issues (review items 1-5) and was subsequently pilot tested [8]. Seven (Items **2, 5, 6, 7, 8, 12, and 13**) of the 14-item quality checklist were considered 'critical' (See 'Guidance for rating the overall confidence in the results of the survey'). For each result of each survey, we made a quality assessment. All checklist items were collected in Excel spreadsheets.

Appendix Table A 11. Quality checklist for results of surveys on review item (#)*

#	Item	Question
1	No conflict of interest and funding issues regarding review item (#)	Were there no potential conflict of interests or funding issues that could have affected the outcome of review item (#)? Answer: Yes/No/Unclear. If No, give the rationale.
2	Selective (non) reporting regarding review item (#)	Was there no risk of selective (non-) reporting bias regarding review item (#)? For example: (1) non registering or publication of the review protocol (2) incomplete reporting on the outcomes of review item (#) or changes in definitions of this review item or changes in analyses of this review item that were not congruent with those planned. Answer: Yes/No/Unclear. If No, give the rationale.
3	Target population, field, and context for review item (#) defined	Were the target population, field, and context for the survey on review item (#) defined? Answer: Yes/No/Unclear.
4	Sampling for review item (#)	Did each individual in the target population have an equal chance for being selected for the survey on review item (#)? Answer: Yes/No/Unclear.
5	Survey methods for review item (#)**	Were there no survey methods that could have introduced bias, i.e., systematic error in the outcomes of the survey on review item (#)? Answer: Yes/No/Unclear. If No, give rationale how bias was introduced.
6	Responding surveyees for review item (#) defined	Were the characteristics of the responding surveyees on review item (#) defined? Answer: Yes/No/Unclear.
7	Responding surveyees for review item (#) representative of the target population	Were the characteristics of the responding surveyees on review item (#) representative of the target population? Answer: Yes/No/Unclear.
8	Review item (#) defined	Was review item (#) defined? Answer: Yes/No/Unclear.
9	Reporting of survey question to assess review item (#)	Did the survey (or any additional file) report the survey question to assess review item (#)? Answer: Yes/No/Unclear.
10	Validation of the survey question to assess review item (#)	Was the question to assess review item (#) validated (tested) a priori e.g., through pilot testing or used in previous surveys? Answer: Yes/No/Unclear.
11	Reporting of the response rate on item (#)***	Was the initial sample size and the number of questionnaires that answered the question on review item (#) reported? Answer: Yes/No/Unclear. Initial sample size refers to any initial sample size, i.e., N1, N2, N3, N4, or N5

12	Response rate on review item (#)**	Did the magnitude of the response rate on review item (#) or the way the response rate (in the case of a low response rate) was managed provide certainty in the validity of the results on this review item? Answer: Yes/No/Unclear.
13	Sample size on the prevalence of review item (#)**	Was the sample size adequate for the prevalence statistic of review item (#)? Answer: Yes/No/Unclear.
14	Complete reporting of outcome measures on review item (#)	Were the complete outcome measures given for review item (#), i.e., were the numerators and denominators reported? Answer: Yes/No/Unclear.

*We assigned 'Unclear' when too few details were reported in the manuscript or additional files to make a judgment of assigning 'Yes' or 'No'.
**Guidance for addressing this question is reported under here in the section 'Guidance for completing the checklist for surveys on HA issues'.
***Definitions of all samples are given in Tables 3 and 4 of the main manuscript.

Guidance for completing the quality checklist for surveys on HA issues

Answering most items is straightforward. Items that need additional guidance are presented under here.

Item 5. Survey methods

For this item we considered 5 subitems: 1) survey delivery; 2) incentives; 3) timeframe; 4) multiple (desired) submissions by the same surveyee; 5) methods to prevent multiple (undesired) submissions of surveys by the same surveyee. We addressed the signaling questions for each subitems. Based on the answers to these signaling questions we addressed the checklist question. 'No' was assigned when bias could be the result of methodological issues in one or more of these subitems, which could lead to systematic error in the outcomes of the survey. When 'No' was scored we gave the rationale.

Appendix Table A12. Signaling questions for Item 5. Survey methods*

Subitem	Signaling questions
Survey delivery (Subitem 1)	Were the methods of survey delivery reported, e.g., email, post, telephone etc. Answer: Yes/No/Unclear. If Yes, give the type of survey delivery.
Incentives (Subitem 2)	Report whether incentives were given to surveyees to complete the questionnaires. Answer: Yes/No/Unclear. If Yes, give the type of incentives.
Timeframe (Subitem 3)	Report whether the timeframe between the year of publication of a research publication and the date of the survey on this publication was reported. Answer: Yes/No/Unclear. If Yes, give the time frame.
Multiple (desired) submissions of surveys by the same surveyee (Subitem 4)	Report whether the manuscript reported that the surveyee completed more than 1 survey, e.g., when surveyees who have published multiple articles in the eligible time span were asked to submit a questionnaire for each published article. Answer: Yes/No/Unclear. If Yes, report on these multiple submissions.
Methods to prevent multiple (undesired) submissions of surveys by the same surveyee (Subitem 5)	Report whether methods were implemented for preventing the submitting of more than one questionnaire by the same surveyee when he/she was invited to submit only one. Answer: Yes/No/Unclear. If yes, give the methods.

*We assigned ‘Unclear’ when too few details were reported in the manuscript or additional files to make a judgment of assigning ‘Yes’ or ‘No’.

Item 7. Characteristics of responding surveyees representative of the target population

Whether the characteristics of the responding surveyees were representative of the target population could only be assessed when the characteristics of both responders and non-responders were reported. ‘Unclear’ was scored when the characteristics of both categories were not reported or partly reported or when only the characteristics of one of both categories were reported. ‘No’ was scored when characteristics of both responders and non-responders were reported, but the characteristics differed substantially to have introduced risk of bias. A rationale for each ‘no’ scores was given.

Item 12. Response rate

The impact of nonresponse on the results of a survey will be little when response rates are high [7], but low response rates may diminish the validity of a survey’s results. However, ‘Yes’ can still be answered to the question in item 12 when response rates are modest, i.e., when authors can show that non-response was not related to the outcome measured and that the characteristics of responders and non-responders are comparable. Whether and how adjustment weighting was implemented was considered when answering the question of item 12. Adjustment weighting refers to correcting for selective nonresponse. For example, assigning higher weights to underrepresented respondents. In this context we also considered the issue of same surveyees submitting more

than one questionnaire, e.g., when surveyees who have published multiple articles in the eligible time span for the survey were asked to submit a questionnaire for each published article.

Item 13. Sample size

We calculated the required sample size with EpiTools epidemiological calculators based on the identified prevalence and the total sample size [9]. The estimated prevalence was calculated with a 0.95 confidence level (desired precision of estimate 0.05). The sample size used to calculate the prevalence of a review item was considered 'inadequate' when this sample size was smaller than the required sample size (as calculated by the EpiTools software) for this prevalence.

Guidance for rating the overall confidence in the results of a survey

Our rating of the overall confidence in the results of a survey reflects how non-implementation of one or more of these 14 safeguard items might possibly have impacted bias of the results of the survey. Because not all results in the same survey are at risk of the same biases, we rated the overall confidence for each result of each survey separately. Seven (Items 3, 5, 6, 7, 8, 12, and 13) of the 14-item checklist were considered 'critical' for this rating. We adopted the rating scheme reported for the AMSTAR 2 critical appraisal tool [10] to assign ratings of the overall confidence in each result of each survey. Appendix Table A13 presents this rating scheme and is an exact copy of the AMSTAR 2 instrument.

Appendix Table A13. Rating the overall confidence in the results of a survey*

Rating	Description
High	No or one non-critical weakness was scored in the 14-item quality checklist
Moderate	*More than one non-critical weakness** was scored in the 14-item quality checklist
Low	*One critical flaw with or without non-critical weaknesses* was scored in the 14-item quality checklist
Critically low	*More than one critical flaw with or without non-critical weaknesses* was scored in the 14-item quality checklist

*Multiple non-critical weaknesses may diminish confidence in the results of a survey and it may be appropriate to move the overall appraisal down from moderate to low confidence

Appendix Table A14. Tabular presentation of the scores of the 14-item quality checklist for review item (#)* **

Reference	Survey question	Item 1	Item 2	Item 3	Item 4	Item 5	Item 6…. Item 14	Overall confidence in the result

* All critical appraisal scores Yes/No/Unclear will be given for each result

** Overall confidence ratings are: High, Moderate, Low, and Critically low

Additional item F Occurrence measures and synthesis methods

Defining outcomes

The definitions of all outcomes and the respective numerators and denominators are presented in Appendix Table A15.

Appendix Table A15. Definition of response rates and primary and secondary outcomes

Outcome	Definition
Number of emails with questionnaires on HA issues sent (N1)	The total number of emails with questionnaires on HA issues sent.
Number of emails with questionnaires on HA issues not bounced (N2)	The total number of emails with questionnaires on HA issues sent that had surveyees with valid email addresses.
Number of questionnaires for which the surveyee was available (N3)	The total number of emails with questionnaires sent to assess HA issues with surveyees with valid email addresses and for which the surveyee was available. Unavailability can be the result of, e.g., automated responses such as 'out of office', 'study leave', 'on strike', 'vacation leave', 'maternity leave' etc.
Number of partly or completely answered questionnaires (N4)	The total number of questionnaires on HA issues received back in which the questions were answered (either partial or completely).
Number of completely answered questionnaires (N5)	The total number of questionnaires on HA issues received back in which all questions were answered.
Overall response rates in questionnaires on HA issues	N4 or N5/N1, N2, or N3
Number of questionnaires that answered the question on review item 1* (N6)	The total number of questionnaires received back in which the question on review item 1* was answered.
Response rate on review item 1*	N6/N1, N2, N3, N4 or N5
Number of questionnaires in which the respondents reported review item 1* (N7)	The number of questionnaires in which the respondents reported review item 1*, i.e., perceiving other co-author(s) as honorary author(s) on a publication.
Prevalence of review item 1* **(Primary outcome)**	N7/N6
Number of questionnaires that answered the question on review item 2** (N8)	The total number of questionnaires received back in which the question on review item 2** was answered.
Response rate on review item 2**	N8/N1, N2, N3, N4, or N5.
Number of questionnaires in which the respondents reported review item 2** (N9)	The number of questionnaires in which the respondents reported review item 2**, i.e., having been approached by others to include honorary author(s) on a publication.
Prevalence of review item 2** **(Primary outcome)**	N9/N8
Number of questionnaires that answered the question on review item 3*** (N10)	The total number of questionnaires received back in which the question on review item 3*** was answered.
Response rate on review item 3***	N10/N1, N2, N3, N4 or N5
Number of questionnaires in which the respondents reported review item 3*** (N11)	The number of questionnaires in which the respondents reported review item 3***, i.e., admitting being an honorary author on a publication.
Prevalence of review item 3*** **(Secondary outcome)**	N11/N10
Number of questionnaires that answered the question on review item 4**** (N12)	The total number of questionnaires received back in which the question on review item 4**** was answered.
Response rate on review item 4****	N12/N1, N2, N3, N4, or N5.
Number of questionnaires in which the respondents reported review item 4**** (N13)	The number of questionnaires in which the respondents reported review item 4****, i.e., admitting adding an honorary author(s) on a publication.
Prevalence of review item 4**** **(Secondary outcome)**	N13/N12
Number of questionnaires that answered the question on review item 5***** (N14)	The total number of questionnaires received back in which the question on review item 5***** was answered.
Response rate on review item 5*****	N14/N1, N2, N3, N4 or N5
Number of questionnaires in which the respondents reported review item 5***** (N15)	The number of questionnaires in which the respondents reported review item 5*****, i.e., admitting having approached others to include honorary author(s) on a publication.
Prevalence of review item 5***** **(Secondary outcome)**	N15/N14

* Review item 1: Researchers perceiving other co-author(s) as honorary author(s) on a publication
** Review item 2: Researchers having been approached by others to include honorary author(s) on a publication
*** Review item 3: Researchers admitting being an honorary author on a publication
**** Review item 4: Researchers admitting adding an honorary author(s) on a publication
*****Review item 5: Researchers admitting having approached others to include honorary author(s) on a publication

Criteria for a quantitative synthesis

As reported in our protocol [8] we planned to refrain from meta-analysis in the following scenarios: (1) less than 2 included surveys (2) very different definitions of outcomes (3) incomplete reporting of proportions (4) biased evidence such as 'Low', and 'Critically low' ratings of the overall confidence in the results of the survey (5) explained and unexplained heterogeneity [1]. In our protocol we also stated that we considered a I^2 larger than 50% as an approximate rule of thumb for not conducting meta-analysis. When applying this rule, we considered that the value of I^2 depends on the direction and magnitude of the outcomes and the strengths of the evidence for the identified heterogeneity [1]. Prior to refraining from meta-analysis, we assessed if solutions were possible for dealing with one or more of these limiting criteria [11,12]. Post hoc changes in the implementation of the criteria for undertaking meta-analyses were reported with rationale.

Investigation of heterogeneity and sensitivity analyses

We only conducted subgroup analyses and meta-regression when at least ten observations on a potentially explanatory variable were reported (i.e., 10 studies that reported data regarding a specific explanatory variable) [1,13] and at least one of these observations differed from the other observations. For the tests of subgroup differences, we reported the value of chi-square (Q), the degrees of freedom (df), and the p value. A p value of < 0.05 was considered to be statistically significant.

For the meta-regression we reported the regression coefficient, the 95% confidence intervals, and the p value, e.g., (regression coefficient, XX, 95%CI: XX to XX, p=XX). During the review process we also assessed specific issues to explore in sensitivity analyses, e.g., the impact of the quality or the characteristics of the survey design of certain reviews on the results of this systematic review [8].

Additional item G (Non) reporting bias assessment

Cochrane suggests using the term non-reporting biases over reporting biases [14]. Non-reporting biases lead to bias due to missing results [14]. We used various strategies to address these biases such as:

1) Using a broad-spectrum search strategy with a high sensitivity.
2) Assessing the availability of registered or published protocols and if available we assessed whether the planned outcomes were the same as those reported in the completed surveys.
3) Contacting of authors to obtain information on possible multiple publication of the same research data, the availability of unpublished, ongoing surveys, and protocols.
4) We implemented the 6-step framework suggested by Cochrane to assess risk of bias due to missing results in a synthesis [14].

We did not conducted tests for funnel plot asymmetry, because there is no evidence that proportional data adequately adjust for these graphical tests [15].

Additional item H Guidance for grading the certainty or quality of evidence for a review item

We used the GRADE approach [16] for grading the certainty or quality of evidence for each outcome of our planned systematic review on survey research. The GRADE approach assigns four levels of certainty: 'High', 'Moderate', 'Low', and 'Very low certainty' that a point estimate for a specific outcome is correct [16]. The rationale for assigning these ratings for each outcome was given.

GRADE ratings for outcomes of interventional studies start with assigning high quality to randomized controlled trials and low quality to observational studies. For surveys we assigned high quality when surveyees had an equal chance of being selected for the survey and low quality when they did not. We started with this initial quality rating and then according to the GRADE approach assessed 5 factors that can lower the quality rating. These 5 factors are presented as domains 1-5 and are explained.

Domain 1. Risk of bias

Domain 2. Inconsistency

Domain 3. Indirectness

Domain 4. Imprecision

Domain 5. Publication bias

Domain 1. Bias in the included surveys.

Bias in the results of the included surveys was based on our 14-item quality checklist reported in Additional item E. For each outcome we assigned one of the following overall confidence ratings: High, Moderate, Low, and Critically low. (See quality checklist for surveys on HA items in Additional item E). The rationale for assigning each type of rating was given for each outcome.

Domain 2. Heterogeneity or inconsistency of results.

We assessed the presence and the extent of heterogeneity. In the forest plots we assessed the overlap of the confidence intervals for the results of the individual surveys. We calculated Tau^2 (Estimate of

between study variance) and Chi^2 tests to measure statistical heterogeneity [1]. We calculated I^2 to quantify inconsistency and used the following rough interpretation of pertinent thresholds for I^2 [1].

0% to 40%**:** might not be important

30% to 60%: may represent moderate heterogeneity

50% to 90%: may represent substantial heterogeneity

75% to 100%: considerable heterogeneity

Domain 3. Indirectness of evidence

Indirectness of evidence was assigned when for example not all, but only a subgroup of corresponding authors (for example only the heads of departments) of a target population were surveyed. Outcomes for such a subgroup were not considered representative for all corresponding authors of that target population.

Domain 4. Imprecision of results

Surveys with few surveyees or with few events are imprecise and have wide confidence intervals [16]. We assessed imprecision for each outcome.

Domain 5. Publication bias

For this domain we assessed whether publication bias is likely. Methods to detect publication bias were reported in the Appendix (Additional Item G, page 17 and Additional Item N, pages 44-45).

Results

Additional item I. Included surveys and characteristics of included surveys

Included surveys

The 19 eligible surveys are reported in Appendix Table A16.

Appendix Table A16. Included surveys

First author/year of publication	Full reference
Al-Herz 2014	Al-Herz W, Haider H, Al-Bahhar M, Sadeq A. Honorary authorship in biomedical journals: how common is it and why does it exist? J Med Ethics. 2014 May;40(5):346-8. doi: 10.1136/medethics-2012-101311. Epub 2013 Aug 17. PMID: 23955369.
Bonekamp 2012	Bonekamp S, Halappa VG, Corona-Villalobos CP, Mensa M, Eng J, Lewin JS, Kamel IR. Prevalence of honorary coauthorship in the American Journal of Roentgenology. AJR Am J Roentgenol. 2012 Jun;198(6):1247-55. doi: 10.2214/AJR.11.8253. PMID: 22623536.
Eisenberg 2011	Eisenberg RL, Ngo L, Boiselle PM, Bankier AA. Honorary authorship in radiologic research articles: assessment of frequency and associated factors. Radiology. 2011 May;259(2):479-86. doi: 10.1148/radiol.11101500. Epub 2011 Mar 8. PMID: 21386051.
Eisenberg 2014	Eisenberg RL, Ngo LH, Bankier AA. Honorary authorship in radiologic research articles: do geographic factors influence the frequency? Radiology. 2014 May;271(2):472-8. doi: 10.1148/radiol.13131710. Epub 2013 Nov 27. PMID: 24475845.
Eisenberg 2018	Eisenberg RL, Ngo LH, Heidinger BH, Bankier AA. Honorary Authorship in Radiologic Research Articles: Assessment of Pattern and Longitudinal Evolution. Acad Radiol. 2018;25(11):1451–1456. doi:10.1016/j.acra.2018.02.023.
Gadjradj 2018	Gadjradj PS, Fezzazi RE, Meppelder CA, Rietdijk WJ, Matabadal NN, Verhemel A, Harhangi BS. Letter: Honorary Authorship in Neurosurgical Literature: A Cross-sectional Analysis. Neurosurgery. 2018 Jan 1;82(1):E25-E28. doi: 10.1093/neuros/nyx525. PMID: 29053850.
Gadjradj 2020	Gadjradj PS, Peul WC, Jalimsing M, Arjun Sharma JRJ, Verhemel A, Harhangi BS. Who should merit co-authorship? An analysis of honorary authorships in leading spine dedicated journals. Spine J. 2020 Jan;20(1):121-123. doi: 10.1016/j.spinee.2019.08.008. Epub 2019 Aug 21. PMID: 31445166.
Gadjradj 2021	Gadjradj PS, Jalimsing M, Jalimsing S, Voigt I. Authorship in Oral and Maxillofacial Surgery. J Maxillofac Oral Surg. 2021 Jun;20(2):330-335. doi: 10.1007/s12663-021-01538-9. Epub 2021 Mar 16. PMID: 33911405; PMCID: PMC8041930.
Gülen 2020	Gülen S, Fonnes S, Andresen K, Rosenberg J. More than one-third of Cochrane reviews had gift authors, whereas ghost authorship was rare. J Clin Epidemiol. 2020 Dec;128:13-19. doi: 10.1016/j.jclinepi.2020.08.004. Epub 2020 Aug 8. PMID: 32781115.
Hardjosantoso 2020	Hardjosantoso HC, Dahi Y, Verhemel A, Dahi I, Gadjradj PS. Honorary Authorships in the Ophthalmological Literature. J Curr Ophthalmol. 2020 Apr 30;32(2):199-202. doi: 10.4103/JOCO.JOCO_104_20. PMID: 32671306; PMCID: PMC7337016.
Ilakovac 2007	Ilakovac V, Fister K, Marusic M, Marusic A. Reliability of disclosure forms of authors' contributions. CMAJ. 2007 Jan 2;176(1):41-6. doi: 10.1503/cmaj.060687. PMID: 17200389; PMCID: PMC1764586.
Kayapa 2018	Kayapa B, Jhingoer S, Nijsten T, Gadjradj PS. The prevalence of honorary authorship in the dermatological literature. Br J Dermatol. 2018 Jun;178(6):1464-1465. doi: 10.1111/bjd.16678. Epub 2018 May 12. PMID: 29663321.
Luiten 2019	Luiten JD, Verhemel A, Dahi Y, Luiten EJT, Gadjradj PS. Honorary Authorships in Surgical Literature. World J Surg. 2019;43(3):696–703. doi:10.1007/s00268-018-4831-3.
Matawlie 2021	Matawlie RH, Arjun Sharma JR, de Rooij JD, Sardjoe Mishre G, Huygen FJ, Gadjradj PS. Honorary authorship in high-impact journals in anaesthesia and pain medicine. Br J Pain. 2021 Aug;15(3):246-248. doi: 10.1177/20494637211023526. Epub 2021 Jun 17. PMID: 34377454; PMCID: PMC8339945.
McClellan 2019	McClellan JM, Mansukhani N, Moe D, Derickson M, Chiu S, Kibbe MR, Martin MJ. Courtesy Authorship in Academic Surgery Publications. JAMA Surg. 2019 Dec 1;154(12):1110-1116. doi: 10.1001/jamasurg.2019.3140. PMID: 31532464; PMCID: PMC6752091.
Noruzi 2019	Noruzi A, Takkenberg JJM, Kayapa B, Verhemel A, Gadjradj PS. Honorary authorship in cardiothoracic surgery [published online ahead of print, 2019 Nov 9]. J Thorac Cardiovasc Surg. 2019;S0022-5223(19)32489-4. doi:10.1016/j.jtcvs.2019.10.104.
Nurmohamed 2021	Nurmohamed FRH, Voigt I, Awadpersad P, Matawlie RHS, Gadjradj PS. Authorship decision-making in the field of orthopedic surgery and sports medicine. J Clin Orthop Trauma. 2021 Jul 28;21:101531. doi: 10.1016/j.jcot.2021.101531. PMID: 34405087; PMCID: PMC8348525.
Rajasekaran 2014	Rajasekaran S, Shan RL, Finnoff JT. Honorary authorship: frequency and associated factors in physical medicine and rehabilitation research articles. Arch Phys Med Rehabil. 2014;95(3):418–428. doi:10.1016/j.apmr.2013.09.024.
Shah 2018	Shah A, Rajasekaran S, Bhat A, Solomon JM. Frequency and Factors Associated With Honorary Authorship in Indian Biomedical Journals: Analysis of Papers Published From 2012 to 2013. J Empir Res Hum Res Ethics. 2018 Apr;13(2):187-195. doi: 10.1177/1556264617751475. Epub 2018 Jan 18. PMID: 29345178.

Characteristics of included surveys

The characteristics of the 19 included surveys are reported in Appendix Tables A17-19. Nine of the eligible surveys originated in the Netherlands and 11 were conducted on corresponding authors. The characteristics of the responding surveyees were reported in all surveys and those of non-responding surveyees were not given in any of the eligible surveys. None of the included surveys reported complete gender/sex breakdowns and only the terms 'males' and 'females' were used [6]. Males were the predominant respondents (>51%) in the 13 of 14 surveys that reported the prevalence of males/females among responding surveyees. The percentage of surveyees that were an associate professor or higher was at least 30% in 10 of the 11 surveys that reported this proportion. Denominators of the different proportions calculated in individual surveys were not always the same, because surveyees did not always respond to all questions of a survey.

Most studies reported a conflict-of-interest statement (17/19) and the limitations of the survey (16/19). In all surveys, participants were sampled consecutively and contacted by email except in one study [17] that did not report their method of survey delivery. In 9 of the included surveys, methods were implemented to avoid the risk of multiple submissions of questionnaires by the same surveyee. In 5 surveys these methods were not implemented and in another 5 surveys nothing was reported on such methods. Incentives to complete the survey were not given in any of the included surveys. In 8 of 18 surveys the time frame between the dates of publication and completing the survey was 1 year or less. The prevalences of the countries or continents of origin of surveyees in the eligible surveys could not be reliably extracted, because of imprecise, partial or non-reporting of this information.

Appendix Table A17. Characteristics of included surveys

Study/year of publication	Number of authors	Country first affiliation	Target field	Target population
Al-Herz 2014	4	Kuwait	Biomedical	Corresponding authors
Bonekamp 2012	7	USA	Radiology	Corresponding authors
Eisenberg 2011	4	USA	Radiology	First authors
Eisenberg 2014	3	USA	Radiology	First authors
Eisenberg 2018	4	USA	Radiology	First authors
Gadjradj 2018	7	The Netherlands	Neurosurgery	Corresponding authors
Gadjradj 2020	7	The Netherlands	Spine	Corresponding authors
Gadjradj 2021	4	The Netherlands	Oral and maxillofacial surgery	Corresponding authors
Gülen 2020	4	Denmark	Cochrane reviews	First authors
Hardjosantoso 2020	5	The Netherlands	Ophtalmology	Corresponding authors
Ilakovac 2007	4	Croazia	Croatian medical journal	Corresponding authors
Kayapa 2018	4	The Netherlands	Dermatology	Corresponding authors
Luiten 2019	5	The Netherlands	General surgery	Corresponding authors
Matawlie 2021	6	The Netherlands	Pain medicine	Mix of corresponding, first, and senior authors
McClellan 2019	7	USA	Surgery	First and senior (last) authors
Noruzi 2019	5	The Netherlands	Cardiothoracic surgery	Corresponding authors
Nurmohamed 2021	5	The Netherlands	Orthopedics and sports medicine	Corresponding authors
Rajasekaran 2014	3	Canada	Physical medicine and rehabilitation	First authors
Shah 2018	4	India	Biomedical	First authors

Appendix Table A18. Characteristics of included surveys

Study/year of publication	Characteristics of the responding surveyees reported	Characteristics of the non-responding surveyees reported	% Males among responding surveyees*	% Females among responding surveyees*	% Associate professor and higher among responding surveyees	Continent of origin of the surveyee**
Al-Herz 2014	Yes	No	75.3% (938/1245)	24.7% (307/1245)	Not assessed	Not assessed
Bonekamp 2012	Yes	No	70.0% (343/490)	30.0% (147/490)	48.8% (239/490)	Not assessed
Eisenberg 2011	Yes	No	76.3% (299/392)	23.7% (93/392)	38.0% (149/392)	Not assessed
Eisenberg 2014	Yes	No	Not reported	Not reported	21.3% (23/108)	Unclear
Eisenberg 2018	Yes	No	Not reported	Not reported	32.0% (73/228)	Unclear
Gadjradj 2018	Yes	No	88.4% (313/354)	11.6% (41/354)	51.7% (193/373)	Unclear
Gadjradj 2020	Yes	No	80.4 (229/285)	19.6% (56/285)	Not assessed	Unclear
Gadjradj 2021	Yes	No	74.9% (170/227)	25.1% (57/227)	Not assessed	Unclear
Gülen 2020	Yes	No	44.6% (297/666)	54.8% (365/666)	32.1% (225/700)	Not assessed
Hardjosantoso 2020	Yes	No	Not reported	Not reported	Not assessed	Unclear
Ilakovac 2007	Yes	No	Not reported	Not reported	Not assessed	Unclear
Kayapa 2018	Yes	No	61.4% (210/342)	38.6% (132/342)	56.2% (187/333)	Unclear
Luiten 2019	Yes	No	77.4% (236/305)	22.6% (69/305)	60.1% (179/298)	Unclear
Matawlie 2021	Yes	No	Not reported	Not reported	Not assessed	Unclear
McClellan 2019	Yes	No	73.4% (271/369)	26.6% (98/369)	Not assessed	Not assessed
Noruzi 2019	Yes	No	86.3% (505/585)	13.7% (80/585)	50.1% (293/585)	Unclear
Nurmohamed 2021	Yes	No	78.3% (375/479)	Not reported	Not assessed	Unclear
Rajasekaran 2014	Yes	No	51.2% (125/244)	48.8% (119/244)	38.2% (71/186)	Unclear
Shah 2018	Yes	No	65.2% (159/244)	34.8% (85/244)	46.5% (67/144)	Unclear

*None of the included surveys gave complete gender/sex breakdowns for all considered categories and only the terms 'males' and 'females' were used [6].
**The continent of origin of the surveyee was either not assessed or Unclear.

Appendix Table A19. Characteristics of included surveys

Study/year of publication	Conflict of interest statement reported	Registration or publication of a protocol	Sampling	Survey delivery	Risk of multiple submissions of surveys by the same surveyee*	Time frame between publishing the manuscript and being surveyed	Incentives given	Limitations reported
Al-Herz 2014	Yes	Not reported	Consecutively	Email	No	≤12 months	Not reported	Not reported
Bonekamp 2012	Not reported	Not reported	Consecutively	Email	No	2-8 years	Not reported	Yes
Eisenberg 2011	Yes	Not reported	Consecutively	Email	No	3 years	Not reported	Yes
Eisenberg 2014	Yes	Not reported	Consecutively	Email	No	2-3 years	Not reported	Yes
Eisenberg 2018	Not reported	Not reported	Consecutively	Email	No	2-3 years	Not reported	Yes
Gadjradj 2018	Yes	Not reported	Consecutively	Email	Unclear	Not reported	Not reported	Not reported
Gadjradj 2020	Yes	Not reported	Consecutively	Email	Unclear	2 years	Not reported	Not reported
Gadjradj 2021	Yes	Not reported	Consecutively	Email	Unclear	1 year	Not reported	Yes
Gülen 2020	Yes	Not reported	Consecutively	Email	No	≥30 months	Not reported	Yes
Hardjosantoso 2020	Yes	Not reported	Consecutively	Email	Yes	1 year	Not reported	Yes
Ilakovac 2007	Yes	Not reported	Consecutively	Not reported	Unclear	At the time of submitting the paper	Not reported	Yes
Kayapa 2018	Yes	Not reported	Consecutively	Email	Yes	1 year	Not reported	Yes
Luiten 2019	Yes	Not reported	Consecutively	Email	No	1 year	Not reported	Yes
Matawlie 2021	Yes	Not reported	Consecutively	Email	Unclear	1 year	Not reported	Yes
McClellan 2019	Yes	Not reported	Consecutively	Email	No	2-3 years	Not reported	Yes
Noruzi 2019	Yes	Not reported	Consecutively	Email	No	1 year	Not reported	Yes
Nurmohamed 2021	Yes	Not reported	Consecutively	Email	Yes	1 year	Not reported	Yes
Rajasekaran 2014	Yes	Not reported	Consecutively	Email	Yes	≤ 3 years and 6 months	Not reported	Yes
Shah 2018	Yes	Not reported	Consecutively	Email	Yes	Not reported	Not reported	Yes

*Submitting multiple surveys is possible when an author had published more than once in any of the eligible journals and completed a survey on each of their published manuscripts. No, is assigned when methods to prevent this were implemented. Unclear, is assigned when methods to prevent this were not reported.

Additional item J. Excluded studies with rationale

Appendix Table A20. Excluded studies with rationale

Reference	Year	Rationale for exclusion after full text assessment
Slone RM. Coauthors' contributions to major papers published in the AJR: frequency of undeserved coauthorship. AJR Am J Roentgenol. 1996 Sep;167(3):571-9. doi: 10.2214/ajr.167.3.8751654. PMID: 8751654.	1996	Did not assess HA issues as defined in our protocol
Rajasekaran S, Lo A, Aly AR, Ashworth N. Honorary authorship in postgraduate medical training. Postgrad Med J. 2015 Sep;91(1079):501-7. doi: 10.1136/postgradmedj-2015-133493. Epub 2015 Aug 25. PMID: 26306503.	2015	Did not assess HA issues as defined in our protocol
Derickson M, McClellan JM, Mansukhani NA, Kibbe MR, Martin MJ. Variations in Courtesy Authorship Perceptions and Practices Among Modern Surgical Journals: The Generation Gap. J Surg Res. 2020 Oct;254:242-246. doi: 10.1016/j.jss.2020.04.034. Epub 2020 May 29. PMID: 32480067.	2020	Did not assess HA issues as defined in our protocol
Ashkenazi I, Olsha O. Honorific authorship and approval of the ICMJE criteria: A survey with a convenience sample. Learned publishing. 2021;34(4):647-654.	2021	Did not assess HA issues as defined in our protocol
Justin GA, Miller SC, Tsou B, Li X, Purt B, Fliotsos MJ, Zhao J, Gardner SE, Legault GL, Yonekawa Y, Rapuano CJ, Woreta FA, Pelton RW. Ghost and Honorary Authorship in Ophthalmology: A Cross-Sectional Survey. Am J Ophthalmol. 2022 Aug;240:67-78. doi: 10.1016/j.ajo.2022.02.012. Epub 2022 Feb 25. PMID: 35227695.	2022	Did not assess HA issues as defined in our protocol
Kwee TC, Almaghrabi M, Kwee RM. Scientific fraud, publication bias, and honorary authorship in nuclear medicine. J Nucl Med. 2022 Sep 8:jnumed.122.264679. doi: 10.2967/jnumed.122.264679. Epub ahead of print. PMID: 36215567.	2022	Did not assess HA issues as defined in our protocol
Wislar JS, Flanagin A, Fontanarosa PB, Deangelis CD. Honorary and ghost authorship in high impact biomedical journals: a cross sectional survey. BMJ. 2011 Oct 25;343:d6128. doi: 10.1136/bmj.d6128. PMID: 22028479; PMCID: PMC3202014.	2011	Did not assess HA issues as defined in our protocol
Aldughmi M, Qutaishat D, Karasneh R. Knowledge and Perceptions of Honorary Authorship among Health Care Researchers: Online Cross-sectional Survey Data from the Middle East. Sci Eng Ethics. 2021 Jun 7;27(3):39. doi: 10.1007/s11948-021-00317-6. PMID: 34100137.	2021	Non eligible participants
Kennedy MS, Barnsteiner J, Daly J. Honorary and ghost authorship in nursing publications. J Nurs Scholarsh. 2014 Nov;46(6):416-22. doi: 10.1111/jnu.12093. Epub 2014 Jun 13. PMID: 24930670.	2014	Did not assess HA issues as defined in our protocol
Condron ME, Kibbe MR, Azarow KS, Martin MJ. Courtesy Authorship Practices Among First and Senior Authors: Evaluation of Motivations, Gender Bias, and Inequities. Ann Surg. 2021 Sep 1;274(3):434-440. doi: 10.1097/SLA.0000000000004999. PMID: 34132701.	2021	Did not assess HA issues as defined in our protocol
Vinther S, Rosenberg J. Appearance of ghost and gift authors in Ugeskrift for Læger and Danish Medical Journal. Dan Med J. 2012 May;59(5):A4455. PMID: 22549492.	2012	We were unable to obtain the survey questions. Excluded, because the authors did not respond to 2 emails to get the questionnaires. Both emails were sent in May 2022. One on May 10 and repeated on May 21.
Hadji M, Asghari F, Yunesian M, Kabiri P, Fotouhi A. Assessing the Prevalence of Publication Misconduct among Iranian Authors Using a Double List Experiment. Iran J Public Health. 2016 Jul;45(7):897-904. PMID: 27516996; PMCID: PMC4980344.	2016	Did not assess HA issues as defined in our protocol
Mowatt G, Shirran L, Grimshaw JM, et al. Prevalence of honorary and ghost authorship in Cochrane reviews. JAMA. 2002;287:2769-2771.	2002	Did not assess HA issues as defined in our protocol
Dotson B, Slaughter RL. Prevalence of articles with honorary and ghost authors in three pharmacy journals. Am J Health Syst Pharm.	2011	Did not assess HA issues as defined in our protocol

2011 Sep 15;68(18):1730-4. doi: 10.2146/ajhp100583. PMID: 21880889.		
Ivaniš A. Značajke autorstva znanstvenih članaka u akademskoj medicini. 2010	2010	Did not assess HA issues as defined in our protocol
Ivanis A, Hren D, Sambunjak D, Marusić M, Marusić A. Quantification of authors' contributions and eligibility for authorship: randomized study in a general medical journal. J Gen Intern Med. 2008;23(9):1303-1310. doi:10.1007/s11606-008-0599-8	2008	Did not assess HA issues as defined in our protocol
Shapiro DW, Wenger NS, Shapiro MF. The contributions of authors to multiauthored biomedical research papers. JAMA. 1994 Feb 9;271(6):438-42. PMID: 8295318.	1994	Did not assess HA issues as defined in our protocol
Masic I. The Malversations of Authorship - Current Status in Academic Community and How to Prevent It. Acta Inform Med. 2018;26(1):4-9. doi: 10.5455/aim.2018.26.4-9. PMID: 29719305; PMCID: PMC5869232.	2018	Did not assess HA issues as defined in our protocol
Flanagin A, Carey LA, Fontanarosa PB, Phillips SG, Pace BP, Lundberg GD, Rennie D. Prevalence of articles with honorary authors and ghost authors in peer-reviewed medical journals. JAMA. 1998 Jul 15;280(3):222-4. doi: 10.1001/jama.280.3.222. PMID: 9676661.	1998	Did not assess HA issues as defined in our protocol
Ivaniš A, Hren D, Marušić M, Marušić A. Less work, less respect: authors' perceived importance of research contributions and their declared contributions to research articles. PLoS One. 2011;6(6):e20206. doi: 10.1371/journal.pone.0020206. Epub 2011 Jun 21. PMID: 21713036; PMCID: PMC3119662.	2011	Did not assess HA issues as defined in our protocol
Okonta P, Rossouw T. Prevalence of scientific misconduct among a group of researchers in Nigeria. Dev World Bioeth. 2013 Dec;13(3):149-57. doi: 10.1111/j.1471-8847.2012.00339.x. Epub 2012 Sep 20. PMID: 22994914; PMCID: PMC3530634.	2013	Did not assess HA issues as defined in our protocol
Artino AR Jr, Driessen EW, Maggio LA. Ethical Shades of Gray: International Frequency of Scientific Misconduct and Questionable Research Practices in Health Professions Education. Acad Med. 2019 Jan;94(1):76-84. doi: 10.1097/ACM.0000000000002412. PMID: 30113363.	2019	Did not assess HA issues as defined in our protocol
Hoen WP, Walvoort HC, Overbeke AJ. What are the factors determining authorship and the order of the authors' names? A study among authors of the Nederlands Tijdschrift voor Geneeskunde (Dutch Journal of Medicine). JAMA. 1998 Jul 15;280(3):217-8. doi: 10.1001/jama.280.3.217. PMID: 9676659.	1998	We were unable to obtain the survey questions through contacting of the authors. No reference or link to questionnaire was given.
Marusic A, Bates T, Anic A, Marusic M (2006) How the structure of contribution disclosure statements affects validity of authorship: a randomized study in a general medical journal. Curr Med Res Opin 22: 1035–44.	2006	Did not assess HA issues as defined in our protocol
Swank JM, Houseknecht A, Puig A, Authorship Decision-Making: A National Survey of Counselor Educators. JCPS 2019;12(2)	2019	Did not assess HA issues as defined in our protocol
Rees CA, Keating EM, Dearden KA, Haq H, Robison JA, Kazembe PN, Bourgeois FT, Niescierenko M. Importance of authorship and inappropriate authorship assignment in paediatric research in low- and middle-income countries. Trop Med Int Health. 2019 Oct;24(10):1229-1242. doi: 10.1111/tmi.13295. Epub 2019 Aug 21. PMID: 31374140.	2019	Did not assess HA issues as defined in our protocol
Chambers LM, Watson CH, Yao M, Levinson K, Alvarez RD, Eskander RN, Buechel M, Michener CM, Jernigan A. Survey of trends in authorship assignment in gynecologic oncology: Keeping score and playing fair. Gynecol Oncol Rep. 2021 Mar 23;36:100755. doi: 10.1016/j.gore.2021.100755. PMID: 33855146; PMCID: PMC8027688.	2021	Did not assess HA issues as defined in our protocol

Kwee RM, Almaghrabi MT, Kwee TC. Scientific integrity and fraud in radiology research. Eur J Radiol. 2022 Oct 8;156:110553. doi: 10.1016/j.ejrad.2022.110553. Epub ahead of print. PMID: 36228454.	2022	Did not assess HA issues as defined in our protocol
Smith E, Williams-Jones B, Master Z, Larivière V, Sugimoto CR, Paul-Hus A, Shi M, Resnik DB. Misconduct and Misbehavior Related to Authorship Disagreements in Collaborative Science. Sci Eng Ethics. 2020 Aug;26(4):1967-1993. doi: 10.1007/s11948-019-00112-4. Epub 2019 Jun 3. PMID: 31161378; PMCID: PMC6888995.	2020	Did not assess HA issues as defined in our protocol
Dhaliwal U, Singh N, Bhatia A. Awareness of authorship criteria and conflict: survey in a medical institution in India. MedGenMed. 2006 Dec 12;8(4):52. PMID: 17415332; PMCID: PMC1868341.	2006	Did not assess HA issues as defined in our protocol
Badreldin H, Aloqayli S, Alqarni R, Alyahya H, Alshehri A, Alzahrani M, Al Tawalbeh A, Ismail WW. Knowledge and Awareness of Authorship Practices Among Health Science Students: A Cross-Sectional Study. Adv Med Educ Pract. 2021 Apr 20;12:383-392. doi: 10.2147/AMEP.S298645. PMID: 33907488; PMCID: PMC8069121.	2021	Did not assess HA issues as defined in our protocol
Nylenna M, Fagerbakk F, Kierulf P. Authorship: attitudes and practice among Norwegian researchers. BMC Med Ethics. 2014 Jul 2;15:53. doi: 10.1186/1472-6939-15-53. PMID: 24989359; PMCID: PMC4118778.	2014	Did not assess HA issues as defined in our protocol
Patience GS, Galli F, Patience PA, Boffito DC. Intellectual contributions meriting authorship: Survey results from the top cited authors across all science categories. PLoS One. 2019 Jan 16;14(1):e0198117. doi: 10.1371/journal.pone.0198117. PMID: 30650079; PMCID: PMC6334927.	2019	Did not assess HA issues as defined in our protocol
Pulsipher KJ, Presley CL, Szeto MD, Barber C, Rietcheck HR, Meckley AL, Militello M, Runion TM, Rundle CW, Dellavalle RP. A survey of osteopathic physician and student authorship in the dermatology literature. Dermatol Online J. 2021 Mar 15;27(3):13030/qt53w5s2vs. PMID: 33865292.	2021	Did not assess HA issues as defined in our protocol
Helgesson G, Juth N, Schneider J, Lövtrup M, Lynøe N. Misuse of Coauthorship in Medical Theses in Sweden. J Empir Res Hum Res Ethics. 2018 Oct;13(4):402-411. doi: 10.1177/1556264618784206. Epub 2018 Jul 9. PMID: 29985088.	2019	Did not assess HA issues as defined in our protocol
Ghajarzadeh M. Guest authors in an Iranian journal. Dev World Bioeth. 2014 Apr;14(1):15-9. doi: 10.1111/dewb.12002. Epub 2012 Oct 1. PMID: 23025813.	2014	Did not assess HA issues as defined in our protocol
Ljubenković AM, Borovečki A, Ćurković M, Hofmann B, Holm S. Survey on the Research Misconduct and Questionable Research Practices of Medical Students, PhD Students, and Supervisors at the Zagreb School of Medicine in Croatia. J Empir Res Hum Res Ethics. 2021 Oct;16(4):435-449. doi: 10.1177/15562646211033727. Epub 2021 Jul 26. PMID: 34310249.	2021	Did not assess HA issues as defined in our protocol
Landa-Blanco M, Santos-Midence C, Landa Blanco A, Academic integrity: attitudes and practices of students of a public university in Honduras. Academia. 2020; Vol 20-21:202-2017.	2020	Did not assess HA issues as defined in our protocol
Osareh F, Serati Shirazi M, Khademi R. A Survey on Co-authorship Network of Iranian Researchers in the field of Pharmacy and Pharmacology in Web of Science during 2000-2012. jha 2014; 17 (56) :33-45	2014	Did not assess HA issues as defined in our protocol
Tarnow E, De Young BR, Cohen MB. Coauthorship in pathology, a comparison with physics and a survery-generated and member-preferred authorship guideline. MedGenMed. 2004 Jul 22;6(3):1-2. PMID: 15520623; PMCID: PMC1435638.	2004	Did not assess HA issues as defined in our protocol
Mitcheson H, Collings S, Siebers RW. Authorship issues at a New Zealand academic institution. Int J Occup Environ Med. 2011 Jul;2(3):166-71. PMID: 23022833.	2011	Did not assess HA issues as defined in our protocol

Kratz JE, Strasser C. Researcher perspectives on publication and peer review of data. PLoS One. 2015 Feb 23;10(2):e0117619. doi: 10.1371/journal.pone.0117619. Erratum in: PLoS One. 2015;10(4):e0123377. PMID: 25706992; PMCID: PMC4338305.	2015	Did not assess HA issues as defined in our protocol
Dhingra D, Mishra D. Publication misconduct among medical professionals in India. Indian J Med Ethics. 2014 Apr 1;11(2):104-7. doi: 10.20529/IJME.2014.026. PMID: 24727622.	2014	Did not assess HA issues as defined in our protocol
Pupovac V, Prijić-Samaržija S, Petrovečki M. Research Misconduct in the Croatian Scientific Community: A Survey Assessing the Forms and Characteristics of Research Misconduct. Sci Eng Ethics. 2017 Feb;23(1):165-181. doi: 10.1007/s11948-016-9767-0. Epub 2016 Mar 3. PMID: 26940319.	2017	Did not assess HA issues as defined in our protocol
Jacard M, Herskovic V, Hernandez I, Reyes H. An analysis of authorship in articles published in Revista Medica de Chile [Spanish]. Rev Med Chil 2002;130:1391-8.	2002	Did not assess HA issues as defined in our protocol
Reinisch JF, Li WY, Yu DC, Walker JW. Authorship conflicts: a study of awareness of authorship criteria among academic plastic surgeons. Plast Reconstr Surg. 2013 Aug;132(2):303e-310e. doi: 10.1097/PRS.0b013e3182958b5a. PMID: 23897358.	2013	Did not assess HA issues as defined in our protocol
Goodman NW. Survey of fulfillment of criteria for authorship in published medical research. BMJ. 1994 Dec 3;309(6967):1482. doi: 10.1136/bmj.309.6967.1482. PMID: 7804054; PMCID: PMC2541657.	1994	Did not assess HA issues as defined in our protocol
Bekkelund SI, Hegstad AC, Førde OH. Uredelighet i medisinsk og helsefaglig forskning i Norge [Scientific misconduct and medical research in Norway]. Tidsskr Nor Laegeforen. 1995 Oct 20;115(25):3148-51. Norwegian. PMID: 8539699.	1995	Did not assess HA issues as defined in our protocol
Rennie D. Freedom and responsibility in medical publication: setting the balance right. JAMA. 1998 Jul 15;280(3):300-2. doi: 10.1001/jama.280.3.300. PMID: 9676691.	1998	Did not assess HA issues as defined in our protocol
Feeser VR, Simon JR. The ethical assignment of authorship in scientific publications: issues and guidelines. Acad Emerg Med. 2008 Oct;15(10):963-9. doi: 10.1111/j.1553-2712.2008.00239.x. Epub 2008 Sep 17. PMID: 18801021.	2008	not a survey
Ivanis A, Hren D, Sambunjak D, Marusić M, Marusić A. Quantification of authors' contributions and eligibility for authorship: randomized study in a general medical journal. J Gen Intern Med. 2008;23(9):1303-1310. doi:10.1007/s11606-008-0599-8	2008	Duplicate. Did not assess HA issues as defined in our protocol
O'Brien J, Baerlocher MO, Newton M, Gautam T, Noble J. Honorary coauthorship: does it matter? Can Assoc Radiol J. 2009 Dec;60(5):231-6. doi: 10.1016/j.carj.2009.09.001. Epub 2009 Oct 9. PMID: 19819102.	2009	Did not assess HA issues as defined in our protocol
Joubert G. Authorship: practices and experiences in the Faculty of Health Sciences of the University of the Free State. SA Fam Pract 2005;47(4): 57-60)	2005	Did not assess HA issues as defined in our protocol

Additional item K. Rating the overall confidence in the results of a survey

The eligible questions gave 51 results of which 45 were included in 4 meta-analyses, because same questions were asked, i.e., Question 1a (n=6 results). Did any of the authors included in the article did not deserve credit for authorship? Question 1b (n=11 results). As a result of your current understanding of ICMJE authorship guidelines, do you believe that any of your coauthors listed for this article did not make sufficient contributions to merit being included as coauthors? Question 1c. (n=15 results). Did any of your co-authors perform only one or more of the following tasks, and nothing else, while working on this Question 2. (n=11 results). Did anyone suggest that you include an 'honorary' author in your manuscript? Question 4a. (n=2) Did you (First author) include an honorary author in your manuscript? The remaining 6 results were obtained from single unique questions that were only asked once and could therefore not be meta-analyzed. The results of the rating of the overall confidence in the results of a survey based on the seven critical items (Items **2, 5, 6, 7, 8, 12, and 13**) of the 14-item quality checklist are presented in Appendix Table A21.

Appendix Table A21. Rating the overall confidence in the results of a survey*

Reference	Review question	Item 2	Item 5	Item 6	Item 7	Item 8	Item 12	Item 13	Overall confidence in the result
Al-Herz 2014	Did any of the authors included in the article did not deserve credit for authorship?	?	?	😊	?	😊	☹	😊	low
Bonekamp 2012	As a result of your current understanding of ICMJE authorship guidelines, do you believe that any of your coauthors listed for this article did not make sufficient contributions to merit being included as coauthors?	?	?	😊	?	😊	☹	😊	low
Bonekamp 2012	Did any of your coauthors perform only tasks from the list below, and nothing else related to the manuscript preparation, study design or data analysis while working on that article?	?	?	😊	?	😊	☹	😊	low
Eisenberg 2011	Do you feel that any of your coauthors in this article did not make sufficient contributions to merit being included as coauthors?	?	?	😊	?	😊	☹	😊	low
Eisenberg 2011	Did any of your coauthors perform only one or more of the following tasks, and nothing else, while working on this article?	?	?	😊	?	😊	☹	☹	critically low
Eisenberg 2014	Do you feel that any of your coauthors in this article did not make sufficient contributions to be included as co-authors?	?	?	😊	?	😊	☹	😊	low
Eisenberg 2014	Did any of your coauthors perform only one or more of the following tasks, and nothing else, while working on this article?	?	?	😊	?	😊	☹	☹	critically low
Eisenberg 2014	Did anyone suggest to include an honorary author in your manuscript?	?	?	😊	?	😊	☹	😊	low

Eisenberg 2018	Do you feel that any of your coauthors in this article did not make sufficient contributions to be included as co-authors?	?	?	😊	?	😊	☹	☹	critically low
Eisenberg 2018	Did any of your coauthors perform only one or more of the following tasks, and nothing else, while working on this article?	?	?	😊	?	😊	☹	☹	critically low
Eisenberg 2018	Did anyone suggest to include an honorary author in your manuscript?	?	?	😊	?	😊	☹	😊	low
Gadjradj 2018	As a result of your current understanding of ICMJE authorship guidelines, do you believe that any of your coauthors listed for this article did not make sufficient contributions to merit being included as coauthors?	?	?	😊	?	😊	☹	😊	low
Gadjradj 2018	Did any of your coauthors perform only one or more of the tasks from the list of the above, and nothing else related to the manuscript preparation, study design or data analysis while working on that article?	?	?	😊	?	😊	☹	☹	critically low
Gadjradj 2018	Did anyone suggest that you include an honorary author?	?	?	😊	?	😊	☹	😊	low
Gadjradj 2020	As a result of your current understanding of ICMJE authorship guidelines, do you believe that any of your coauthors listed for this article did not make sufficient contributions to merit being included as coauthors?	?	?	😊	?	😊	☹	😊	low
Gadjradj 2020	Did any of your coauthors perform only one or more of the tasks from the list of the above, and nothing else related to the manuscript preparation, study design or data analysis while working on that article?	?	?	😊	?	😊	☹	☹	critically low
Gadjradj 2020	Did anyone suggest that you include an honorary author?	?	?	😊	?	😊	☹	😊	low
Gadjradj 2021	As a result of your current understanding of ICMJE authorship guidelines, do you believe that any of your coauthors enlisted for the current article did not make sufficient contributions to merit coauthorship?	?	?	😊	?	😊	☹	😊	low
Gadjradj 2021	Did any of your coauthors performed only one or more ''non-authorship'' tasks and nothing else related to study design, manuscript preparation etc.?	?	?	😊	?	😊	☹	☹	critically low
Gadjradj 2021	Did anyone suggest to include an honorary author?	?	?	😊	?	😊	☹	😊	low
Gülen 2020	Do you (first author) believe that any of your coauthors listed for the review did not make sufficient contributions to merit authorship according to the ICMJE authorship criteria?	?	☹	😊	?	😊	😊	😊	low
Gülen 2020	How many of your coauthors had only one of the following functions, meaning they did only one of these functions and nothing else?	?	☹	😊	?	😊	😊	😊	low
Gülen 2020	Think about your role in the development of the review. Check all the functions you personally performed for the review. First author did not conceive or design the work, conduct literature search, or analyze and interpret data.	?	☹	😊	?	😊	😊	😊	low
Gülen 2020	Think about your role in the development of the review. Check all the functions you personally performed for the review. First author did not draft or revise the review.	?	☹	😊	?	😊	😊	😊	low
Gülen 2020	Think about your role in the development of the review. Check all the functions you personally performed for the review. First author did not give the final approval	?	☹	😊	?	😊	😊	😊	low
Gülen 2020	Did you include a "gift" author in the review? (A gift author is defined as an author, who is listed on the byline but does not meet all authorship criteria from ICMJE.)	?	☹	😊	?	😊	😊	😊	low
Hardjosantoso 2020	As a result of your current understanding of ICMJE authorship guidelines, do you believe that any of your coauthors listed for this article did not make sufficient contributions to merit being included as coauthors?	?	☹	😊	?	😊	☹	😊	critically low
Hardjosantoso 2020	Did any of your coauthors perform only one or more of the tasks from the list of the above, and nothing else	?	☹	😊	?	😊	☹	☹	critically low

	related to the manuscript preparation, study design or data analysis while working on that article?								
Hardjosantoso 2020	Did anyone suggest that you include an honorary author?	?	☹	😊	?	😊	☹	😊	critically low
Ilakovac 2007	We ask authors to specify their individual contributions, following the authorship rules of the Uniform Requirements for Manuscripts Submitted to Medical Journals?	?	?	😊	?	😊	😊	☹	low
Kayapa 2018	As a result of your current understanding of ICMJE authorship guidelines, do you believe that any of your coauthors listed for this article did not make sufficient contributions to merit being included as coauthors?	?	☹	😊	?	😊	☹	😊	critically low
Kayapa 2018	Did any of your coauthors perform only one or more of the tasks from the list of the above, and nothing else related to the manuscript preparation, study design or data analysis while working on that article?	?	☹	😊	?	😊	☹	☹	critically low
Luiten 2019	As a result of your current understanding of ICMJE authorship guidelines, do you believe that any of your coauthors listed for this article did not make sufficient contributions to merit being included as coauthors?	?	?	😊	?	😊	☹	😊	low
Luiten 2019	Did any of your coauthors perform only one or more of the tasks from the list of the above, and nothing else related to the manuscript preparation, study design or data analysis while working on that article?	?	?	😊	?	😊	☹	☹	critically low
Luiten 2019	Did anyone suggest that you include an honorary author?	?	?	😊	?	😊	☹	😊	low
Matawlie 2021	As a result of your current understanding of ICMJE authorship guidelines, do you believe that any of your coauthors listed for this article did not make sufficient contributions to merit being included as coauthors?	?	?	😊	?	😊	☹	😊	low
Matawlie 2021	Did any of your coauthors perform only one or more of the tasks from the list of the above, and nothing else related to the manuscript preparation, study design or data analysis while working on that article?	?	?	😊	?	😊	☹	☹	critically low
Matawlie 2021	Did anyone suggest that you include an honorary author?	?	?	😊	?	😊	☹	😊	low
McClellan 2019	Did you add a courtesy author to the peer-reviewed publication in the journal listed above? (First authors)	?	?	😊	?	😊	☹	☹	critically low
McClellan 2019	Did you add a courtesy author to the peer-reviewed publication in the journal listed above? (Senior, i.e., authors)	?	?	😊	?	😊	☹	☹	critically low
Noruzi 2019	As a result of your current understanding of ICMJE authorship guidelines, do you believe that any of your coauthors listed for this article did not make sufficient contributions to merit being included as coauthors?	?	?	😊	?	😊	☹	😊	low
Noruzi 2019	Did any of your coauthors perform only one or more of the tasks from the list of the above, and nothing else related to the manuscript preparation, study design or data analysis while working on that article?	?	?	😊	?	😊	☹	😊	low
Noruzi 2019	Did anyone suggest that you include an honorary author?	?	?	😊	?	😊	☹	😊	low
Nurmohamed 2021	As a result of your current understanding of ICMJE authorship guidelines, do you believe that any of your coauthors listed for this article did not make sufficient contributions to merit being included as coauthors?	?	☹	😊	?	😊	☹	😊	critically low
Nurmohamed 2021	Did any of your coauthors perform only one or more of the tasks from the list of the above, and nothing else related to the manuscript preparation, study design or data analysis while working on that article?	?	☹	😊	?	😊	☹	☹	critically low
Nurmohamed 2021	Did anyone suggest that you include an honorary author?	?	☹	😊	?	😊	☹	😊	critically low
Rajasekaran 2014	Do you feel that any of your coauthors in this article did not make sufficient contributions to merit being included as coauthors?	?	☹	😊	?	😊	☹	😊	critically low

Rajasekaran 2014	Did any of your coauthors perform only one or more of the following tasks, and nothing else, while working on this article?	?	☹	😊	?	😊	☹	☹	critically low
Rajasekaran 2014	Did anyone suggest that you include an "honorary" author in your manuscript?	?	☹	😊	?	😊	☹	😊	critically low
Shah 2018	Did any of your coauthors not make sufficient contributions to merit being included as a coauthor in your manuscript?	?	☹	😊	?	😊	☹	☹	critically low
Shah 2018	Did any of your coauthors perform only one or more of the following tasks, and nothing else, while working on this article?	?	☹	😊	?	😊	☹	☹	critically low
**Total ?**		**51**	**32**	**0**	**51**	**0**	**0**	**0**	
**Total** 😊		**0**	**0**	**51**	**0**	**51**	**7**	**33**	
**Total** ☹		**0**	**19**	**0**	**0**	**0**	**44**	**18**	

? = unclear risk of bias
😊 = low risk of bias
☹ = high risk of bias

*The quality items scored in Appendix Table A21 "Rating the overall confidence in the results of a survey" are further explained in Additional item E.
Quality item 2: Selective (non) reporting regarding review item (#)
Quality item 5: Risk of bias associated with the survey methods
Quality item 6: Defining the characteristics of the responding surveyees
Quality item 7: Characteristics of the responding surveyees on the review item representative for the target population?
Quality item 8: Defining the review item
Quality item 12: Magnitude of the response rate on the review item
Quality item 13: Magnitude of the sample size

Additional item L. Results of survey questions

Results of multiple surveys using the same research question

Appendix Tables A22-26 present the results of multiple surveys using the same research question. These results were quantitatively synthesized in meta-analyses. The results of these meta-analyses were reported in the main manuscript.

Appendix Table A22. Results for Question 1a. Do you feel that any of your co-authors in this article did not make sufficient contributions to merit being included as co-authors?

Author	Year of publication	Numerator response rate	Denominator response rate (type of denominator)*	Response rate	Numerator review item	Denominator review item	Prevalence review item
Al-Herz	2014	1246	7909 (N2)	0.157542	416	1246	0.333868
Eisenberg	2011	392	1338 (N1)	0.292975	102	392	0.260204
Eisenberg	2014	328	1337 (N2)	0.245325	91	328	0.277439
Eisenberg	2018	309	1839 (N3)	0.168026	97	309	0.313916
Rajasekaran	2014	244	908 (N3)	0.268722	44	244	0.180328
Shah	2018	239	908 (N2)	0.263216	50	239	0.209205

*N1: Number of emails with questionnaires sent, N2: Number of emails with questionnaires not bounced, N3: Number of questionnaires for which the surveyee was available

Appendix Table A23. Results for Question 1b. As a result of your current understanding of ICMJE authorship guidelines, do you believe that any of your coauthors listed for this article did not make sufficient contributions to merit being included as coauthors?

Author	Year of publication	Numerator response rate	Denominator response rate (type of denominator)*	Response rate	Numerator review item	Denominator review item	Prevalence review item
Bonekamp	2012	490	1179 (N2)	0.41560645	121	490	0.246939
Gadjradj	2018	332	1143 (N2)	0.29046369	80	332	0.240964
Gadjradj	2020	285	1180 (N2)	0.24152542	65	285	0.22807
Gadjradj	2021	226	914 (N2)	0.24726477	35	226	0.154867
Gülen	2020	666	1221 (N3)	0.54545455	128	666	0.192192
Hardjosantoso	2020	329	1688 (N1)	0.19490521	29	329	0.088146
Kayapa	2018	343	1359 (N1)	0.25239146	49	343	0.142857
Luiten	2019	307	1037 (N3)	0.29604629	46	307	0.149837
Matawlie	2021	230	1051 (N1)	0.2188392	31	230	0.134783
Noruzi	2019	585	1511 (N3)	0.38716082	148	585	0.252991
Nurmohamed	2021	479	1392 (N2)	0.3441092	70	479	0.146138

*N1: Number of emails with questionnaires sent, N2: Number of emails with questionnaires not bounced, N3: Number of questionnaires for which the surveyee was available

Appendix Table A24. Results for Question 1c. Did any of your co-authors perform only one or more of the following tasks, and nothing else, while working on this article? These tasks refer to: 1. Supervising/recruiting co-authors 2. Obtaining funding or material support 3. Recruiting study subjects 4. Performing cases used in the study 5. Contributing illustrations 6. Reviewing the manuscript 7. Approving manuscript before submission to a journal 8. Signing statement of copyright transfer to journal

Author	Year of publication	Numerator response rate	Denominator response rate (type of denominator)*	Response rate	Numerator review item	Denominator review item	Prevalence review item
Bonekamp	2012	490	1179 (N2)	0.41560645	282	490	0.57551
Eisenberg	2011	392	1338 (N1)	0.29297459	231	392	0.589286
Eisenberg	2014	328	1337 (N2)	0.24532536	165	328	0.503049
Eisenberg	2018	291	1839 (N3)	0.15823817	158	291	0.542955
Gadjradj	2018	332	1143 (N2)	0.29046369	209	332	0.629518
Gadjradj	2020	285	1180 (N2)	0.24152542	140	285	0.491228
Gadjradj	2021	227	914 (N2)	0.24835886	113	227	0.497797
Hardjosantoso	2020	329	1688 (N1)	0.19490521	131	329	0.398176
Kayapa	2018	343	1359 (N1)	0.25239146	142	343	0.413994
Luiten	2019	307	1037 (N3)	0.29604629	135	307	0.439739
Matawlie	2021	230	1051 (N1)	0.2188392	92	230	0.4
Noruzi	2019	585	1511 (N3)	0.38716082	367	585	0.62735
Nurmohamed	2021	479	1392 (N2)	0.3441092	201	479	0.419624
Rajasekaran	2014	248	908 (N3)	0.27312775	137	248	0.552419
Shah	2018	245	908 (N2)	0.26982379	147	245	0.6

*N1: Number of emails with questionnaires sent, N2: Number of emails with questionnaires not bounced, N3: Number of questionnaires for which the surveyee was available

Appendix Table A25. Results for Question 2. Did anyone suggest that you include an 'honorary' author in your manuscript?*

Author	Year of publication	Numerator response rate	Denominator response rate (type of denominator)**	Response rate	Numerator review item	Denominator review item	Prevalence review item
Eisenberg	2014	328	1337 (N2)	0.245325	41	328	0.125
Eisenberg	2018	309	1839 (N3)	0.168026	37	309	0.119741
Gadjradj	2018	354	1143 (N2)	0.309711	30	354	0.084746
Gadjradj	2020	281	1180 (N2)	0.238136	32	281	0.113879
Gadjradj	2021	224	914 (N2)	0.245077	39	224	0.174107
Hardjosantoso	2020	328	1688 (N1)	0.194313	22	328	0.067073
Luiten	2019	303	1037 (N3)	0.292189	27	303	0.089109
Matawlie	2021	226	1051 (N1)	0.215033	21	226	0.09292
Noruzi	2019	583	1511 (N3)	0.385837	66	583	0.113208
Nurmohamed	2021	479	1392 (N2)	0.344109	41	479	0.085595
Rajasekaran	2014	248	908 (N3)	0.273128	23	248	0.092742

* It was not reported whether this outcome referred specifically to perceived or ICMJE-based HA or both.
**N1: Number of emails with questionnaires sent, N2: Number of emails with questionnaires not bounced, N3: Number of questionnaires for which the surveyee was available

Appendix Table A26. Results for Question 4a. Did you (First author) include an honorary author* in your manuscript?

Author	Year of publication	Numerator response rate	Denominator response rate (type of denominator)**	Response rate	Numerator review item	Denominator review item	Prevalence review item
Gülen	2020	666	1221 (N3)	0.545	101	666	0.152
McClellan	2019	157	2222 (N1)	0.071	32	157	0.204

*ICMJE-based honorary authorship
**N1: Number of emails with questionnaires sent, N3: Number of questionnaires for which the surveyee was available

Results of single surveys using a unique research question

Tables A27-32 present the results of single surveys using a unique research question that was not assessed in any other eligible survey.

Appendix Table A27. Results for Question 1d.* How many of your coauthors had only one of the following functions, meaning they did only one of these functions and nothing else? (For each function below, give the number of coauthors, who did only that function). These functions refer to: 1. Conceiving or designing the work 2. Conducting the literature search 3. Analyzing/interpreting literature/data 4. Performing statistical analysis 5. Writing the manuscript or part of the manuscript 6. Revising the manuscript critically for important intellectual content 7. Approving manuscript before submission to a journal 8. Supervising the work or any of the coauthors 9. Recruiting coauthors 10. Communicating with journal editor(s) 11. Obtaining funding or material support 12. Reviewing proofs or the journal's edited version of the review.

Author	Year of publication	Numerator response rate	Denominator response rate (type of denominator)**	Response rate	Numerator review item	Denominator review item	Prevalence review item
Gülen	2020	666	1221 (N3)	54.5%	148	666	22.2%

*The results of single survey question 1d could not be combined with the meta-analyzed results for question 1c, because question 1d did not assess whether there were one or more honorary authors on a publication, but assessed the number of honorary authors in a specific publication.

**N3: Number of questionnaires for which the surveyee was available.

Appendix Table A28. Results for Question 3a.* Think about your role in the development of the review. Check all of the functions you personally performed for the review. Functions: 1. Conceiving or designing the work 2. Conducting the literature search 3. Analyzing/interpreting literature/data 4. Performing statistical analysis 5. Writing the manuscript or part of the manuscript 6. Revising the manuscript critically for important intellectual content 7. Approving manuscript before submission to a journal 8. Supervising the work or any of the coauthors 9. Recruiting coauthors 10. Communicating with journal editor(s) 11. Obtaining funding or material support 12. Reviewing proofs or the journal's edited version of the review. Function checked: First author did not conceive or design the work, conduct literature search, or analyze and interpret data

Author	Year of publication	Numerator response rate	Denominator response rate (type of denominator)**	Response rate	Numerator review item	Denominator review item	Prevalence review item
Gülen	2020	666	1221 (N3)	54.5%	1	666	0.15%

*Results for Questions 3a, 3b, 3c, and 3d could not be quantitatively synthesized because of differences between the included surveyees, different questions and criteria for assigning HA.

**N3: Number of questionnaires for which the surveyee was available.

Appendix Table A29. Results for Question 3b.* Think about your role in the development of the review. Check all of the functions you personally performed for the review. Functions: 1. Conceiving or designing the work 2. Conducting the literature search 3. Analyzing/interpreting literature/data 4. Performing statistical analysis 5. Writing the manuscript or part of the manuscript 6. Revising the manuscript critically for important intellectual content 7. Approving manuscript before submission to a journal 8. Supervising the work or any of the coauthors 9. Recruiting coauthors 10. Communicating with journal editor(s) 11. Obtaining funding or material support 12. Reviewing proofs or the journal's edited version of the review. Function checked: First author did not draft or revise the review.

Author	Year of publication	Numerator response rate	Denominator response rate (type of denominator)**	Response rate	Numerator review item	Denominator review item	Prevalence review item
Gülen	2020	666	1221 (N3)	54.5%	1	666	0.15%

*Results for Questions 3a, 3b, 3c, and 3d could not be quantitatively synthesized because of differences between the included surveyees, different questions and criteria for assigning HA.

**N3: Number of questionnaires for which the surveyee was available

Appendix Table A30. Results for Question 3c.* Think about your role in the development of the review. Check all of the functions you personally performed for the review. Functions: 1. Conceiving or designing the work 2. Conducting the literature search 3. Analyzing/interpreting literature/data 4. Performing statistical analysis 5. Writing the manuscript or part of the manuscript 6. Revising the manuscript critically for important intellectual content 7. Approving manuscript before submission to a journal 8. Supervising the work or any of the coauthors 9. Recruiting coauthors 10. Communicating with journal editor(s) 11. Obtaining funding or material support 12. Reviewing proofs or the journal's edited version of the review. Function checked: First author did not give the final approval.

Author	Year of publication	Numerator response rate	Denominator response rate (type of denominator)**	Response rate	Numerator review item	Denominator review item	Prevalence review item
Gülen	2020	666	1221 (N3)	54.5%	48	666	7.2%

*Results for Questions 3a, 3b, 3c, and 3d could not be quantitatively synthesized because of differences between the included surveyees, different questions and criteria for assigning HA.
**N3: Number of questionnaires for which the surveyee was available.

Appendix Table A31. Results for Question 3d.* In the spaces marked "Contribution Codes," those code letters from the box should be marked that designate substantive contribution(s) of individual authors to the paper. These contribution codes refer to: 1. Conception and design 2. Analysis and interpretation of the data 3. Provision of study materials or patients 4. Collection, assembly and possession of raw data 5. Statistical expertise 6. Drafting of the article 7. Critical revision of the article for important intellectual content 8. Final approval of the article 9. Obtaining of funding 10. Administrative, technical, or logistic support 11. Guarantor of the study 12. Other (specify):

Author	Year of publication	Numerator response rate	Denominator response rate (type of denominator)**	Response rate	Numerator review item	Denominator review item	Prevalence review item
Ilakovac	2007	201	279 (N1)	72%	67	201	33.3%

*Results for Questions 3a, 3b, 3c, and 3d could not be quantitatively synthesized because of differences between the included surveyees, different questions and criteria for assigning HA.
**N1: Number of emails with questionnaires sent

Appendix Table A32. Results for Question 4b. Did you (Senior author, i.e., the last author) add a courtesy author?* **

Author	Year of publication	Numerator response rate	Denominator response rate (type of denominator)***	Response rate	Numerator review item	Denominator review item	Prevalence review item
McClellan	2019	87	2222 (N1)	3.9%	10	87	11.5%

*Courtesy authorship is defined as ICMJE-based honorary authorship
**We did not include the results for question 4b in the meta-analysis of question 4a, because questions 4a and 4b were conducted on the same sample of manuscripts.
**N1: Number of emails with questionnaires sent

Additional item M. Investigation of heterogeneity

Our protocol presented a series of pre-specified explanatory variables to explore heterogeneity through subgroup analyses and meta-regressions. Below we list these variables and explain why certain of these variables were not further explored.

The following pre-specified explanatory variables were explored in meta-regressions:

- Year of the publication of the survey
- Career levels of the survey, i.e., the percentage of respondents being at least associate professors.

The following pre-specified explanatory variables were explored in subgroup analyses:

- The type of authors that were surveyed, i.e., being a corresponding author or not
- Career levels of the survey, i.e., the percentage of respondents being at least associate professors.
- The country of origin of the survey
- The time point for measuring the outcome (the recall period), e.g., before or after 1 year of publication of the manuscript on which the authors were surveyed.

The following additional explanatory variables were explored in subgroup analyses (Reported on pages 2 and 3 of this appendix: Additional item A ‘Differences between the protocol and the final systematic review’)

- Risk of multiple submissions of questionnaires by surveyees
- $\geq$ 50% of the surveyees being a male

The following pre-specified explanatory variables were not explored in either meta-regressions or subgroup analyses. The rationale for these decisions were given for each variable:

- The field of research on which the survey was interviewed. Rationale for exclusion: Less than 10 observations per moderator were reported or the fields of research varied over a wide spectrum.
- The Journal impact factor. Rationale for exclusion: Less than 10 observations per moderator were reported or combinations of different journals were included in the same survey, making the assignment of a specific journal impact factor unreliable.
- The method of survey delivery. Rationale for exclusion: All explanatory variables were the same or were not reported, i.e., the survey delivery was either by email or was not reported (Appendix Table A19).
- Anonymity of the surveyee. Rationale for exclusion: This variable could not be extracted from the included surveys.
- Definitions of honorary authorship. Rationale for exclusion: All meta-analyses were already divided in subgroups according to the definitions of honorary authorship used in the included surveys.
- The magnitude of the response rates. Rationale for exclusion: Different denominators or unclear denominators were used to calculate response rates in the included surveys.
- The method of sampling. Rationale for exclusion: All included surveys used the same method of sampling, i.e., consecutive.
- Study quality: All included surveys were either rated as low or critically low, which are very close ratings based on subjective judgments.

Meta-regression

We conducted meta-regression to assess whether between study heterogeneity could be explained by a series of continuous moderators. Meta-regression was conducted when at least 10 observations per moderator were reported (i.e., 10 studies that reported a specific explanatory moderator) [13] and at least one of these observations differed from the other observations. Meta-regression was not conducted for the findings of review questions 1a and question 4a, because less than 10 surveys were included in the respective meta-analyses. Meta-regression was possible for the results of question 1b, 1c, and 2, because these questions were addressed in 10 or more surveys (Tables 3 and 4). The following explanatory moderators were assessed for these questions: year of publication of the survey and the percentage of respondents being at least associate professors. The results of these analyses and the rationale of non-eligibility of certain explanatory moderators were reported in Appendix Tables A33-35 We reported the regression coefficient, the 95% confidence intervals, and the p value, e.g., (regression coefficient, XX, [95%CI XX to XX], p=XX).

Four meta-regressions could be conducted for the outcomes for questions 1b, 1c, and 2. The prevalence of publications in which researchers perceived that other co-author(s) did not fulfill the ICMJE criteria for authorship decreased with increasing years of publication of the survey (regression coefficient, -0.013, [95%CI: -0.0007 to -0.025], p=0.04) (Appendix Table A34). No associations were found with the other 3 meta-regressions (Appendix Table A33-35).

Appendix Table A33. Question 1b. As a result of your current understanding of ICMJE authorship guidelines, do you believe that any of your coauthors listed for this article did not make sufficient contributions to merit being included as coauthors?

Moderator	Eligible (yes/no)	# of observations	Rationale for non-eligibility	Regression coefficient	95% Confidence Intervals (CI), and p value
Year of publication of the survey	Yes	11		-0.011	95% CI -0.023-0.0010, p = 0.072
Percentage of associate professors and higher among the surveyees	No	6	Less than 10 observations		

Appendix Table A34. Question 1c. Researchers perceiving other co-author(s) as honorary author(s) on a publication based on a list of co-author's contributions. These tasks refer to: 1. Supervising/recruiting co-authors 2. Obtaining funding or material support 3. Recruiting study subjects 4. Performing cases used in the study 5. Contributing illustrations 6. Reviewing the manuscript 7. Approving manuscript before submission to a journal 8. Signing statement of copyright transfer to journal

Moderator	Eligible (yes/no)	# of observations	Rationale for non-eligibility	Regression coefficient	95% Confidence Intervals (CI), and p value
Year of publication of the survey	Yes	15		-0.013	95% CI -0.00066 to -0.025 , p = 0.04*
Percentage of associate professors and higher among the surveyees	Yes	10		-0.092	95% CI -0.53-0.34, p = 0.70

*P<0.05

Appendix Table A35. Question 2. Did anyone suggest that you include an 'honorary' author in your manuscript?

Moderator	Eligible (yes/no)	# of observations	Rationale for non-eligibility	Regression coefficient	95% Confidence Intervals (CI), and p value
Year of publication of the survey	Yes	11		-0.0011	95% CI -0.0076-0.0053, p = 0.73
Percentage of associate professors and higher among the surveyees	No	6	Less than 10 observations		

Subgroup analyses

We conducted subgroup analyses when at least ten observations on potentially explanatory interactions were reported (i.e., 10 or more studies that reported a specific explanatory variable) [1] and when at least one of these observations differed from the other observations. Subgroup analyses were not conducted for the meta-analyzed results of review question 1a and question 4a, because respectively only 6 and 2 surveys were included. Instead, these analyses were possible for questions 1b, 1c, and 2, because they were addressed in at least ten surveys (Tables 3 and 4). We assessed the explanatory variables: 1) being a corresponding author 2) $\geq$50% of the surveyees being male 3) $\geq$50% of the surveyees being an associate professor or higher 4) the presence of risk of multiple submissions by same surveyees 5) the time frame between publication of the manuscript by the surveyee and conducting the survey was $\leq$1 year and 6) whether the survey was conducted by a research group whose' first author was affiliated with a research institute in the Netherlands or not. The results of these analyses and the rationale for non-eligibility of certain explanatory variables were reported in tables. For the tests of group differences, we reported the value of chi-square (Q), the degrees of freedom (df), and the p value. A p value of < 0.05 was considered to be statistically significant. In 8 of the 11 subgroup analyses no significant association was found with the results of the pertinent questions asked (Appendix Tables A36-38). Three subgroup analyses found a significant association ($p<0.05$). When the survey was conducted by a research group whose' first author was affiliated with a research institute in the Netherlands it was less likely ($Q(1) = 5.60$, $p = 0.018$) that researchers perceived other co-author(s) as honorary author(s) on a publication based on a list of co-author's contributions (review question 1c) than when these surveys were conducted in other countries than the Netherlands (Appendix Table A37). When the surveyee was conducted within 1 year or less after the publication of the manuscript, perceived honorary authorship based on ICMJE criteria (Question 1b) and honorary authorship based on researchers' reported contributions of co-authors compared to the ICMJE criteria (Question 1c) were

less likely (respectively Q (1) = 6.62, p = 0.01 for question 1b and (Q (1) = 5.79, p = 0.016 for question 1c) than when these surveys were conducted after more than 1 year (Appendix Tables A36 and 37).

Appendix Table A36. Question 1b. As a result of your current understanding of ICMJE authorship guidelines, do you believe that any of your coauthors listed for this article did not make sufficient contributions to merit being included as coauthors?

**Variable**	**Eligible (yes/no)**	**# of observations**	**Rationale for non-eligibility**	**Test of group differences***
Netherlands country of first affiliation	Yes	11		Q (1) = 2.10, p = 0.147
Corresponding author (target population)	Yes	11		Q (1) = 0.23, p = 0.633
> 50% males among surveyees	No	9	Less than 10 observations	
> 50% associate professor or higher among surveyees	No	6	Less than 10 observations	
Risk of multiple submissions by surveyees	No	7	Less than 10 observations	
Time frame <1 year between publication of manuscript and conducting the survey	Yes	10		Q (1) = 6.62, p = 0.01**

*The chi squared test, 'Q', and the degrees of freedom (df), and the p value 'p'
**P<0.05

Appendix Table A37. Question 1c. Researchers perceiving other co-author(s) as honorary author(s) on a publication based on a list of co-author's contributions. These tasks refer to: 1. Supervising/recruiting co-authors 2. Obtaining funding or material support 3. Recruiting study subjects 4. Performing cases used in the study 5. Contributing illustrations 6. Reviewing the manuscript 7. Approving manuscript before submission to a journal 8. Signing statement of copyright transfer to journal

**Variable**	**Eligible (yes/no)**	**# of observations**	**Rationale for non-eligibility**	**Test of group differences***
Netherlands country of first affiliation	Yes	15		Q (1) = 5.60, p = 0.018**
Corresponding author (target population)	Yes	15		Q (1) = 0.57, p = 0.4449
> 50% males among surveyees	No	11	No differences between observations	
> 50% associate professor or higher among surveyees	Yes	10		Q (1) = 0.30, p = 0.584
Risk of multiple submissions by surveyees	Yes	11		Q (1) = 2.18, p = 0.14
Time frame <1 year between publication of manuscript and conducting the survey	Yes	13		Q (1) = 5.79, p = 0.016**

*The chi squared test, 'Q', and the degrees of freedom (df), and the p value 'p'
**P<0.05

Appendix Table A38. Question 2. Did anyone suggest that you include an 'honorary' author in your manuscript?

Variable	Eligible (yes/no)	# of observations	Rationale for non-eligibility	Test of group differences*
Netherlands country of first affiliation	Yes	11		Q (1) = 1.01, p = 0.315
Corresponding author (target population)	Yes	11		Q (1) = 0.33, p = 0.564
$\geq$ 50% males among surveyees	No	7	Less than 10 observations	
$\geq$ 50% associate professor or higher among surveyees	No	6	Less than 10 observations	
Risk of multiple submissions by surveyees	No	7	Less than 10 observations	
Time frame $\leq$1 year between publication of manuscript and conducting the survey	Yes	10		Q (1) = 0.69, p = 0.407

*The chi squared test, 'Q', and the degrees of freedom (df), and the p value 'p'

Additional item N. Non-reporting biases

Non-reporting biases

Non-reporting biases lead to bias due to missing results in a synthesis of a systematic review [14]. We used the following Cochrane's 6-step framework to explore this type of bias in a synthesis [14]:

Step 1. Select syntheses to assess for risk of bias due to missing results.

We selected all outcomes that were quantitatively synthesized in meta-analyses, i.e., the results to questions 1a, 1b, 1c, 2, and 4.

Step 2. Define which results are eligible for inclusion in each synthesis.

All results to questions 1a, 1b, 1c, 2, and 4 were eligible and non-reporting biases are reported in Appendix Table A39.

Step 3. Record whether any of the studies identified are missing from each synthesis because results known (or presumed) to have been generated by study investigators are unavailable: the 'known unknowns'.

We could not compare the reported results in the surveys with those planned in the protocols, because none of the included surveys registered or published its protocol a priori. We contacted authors of multiple surveys to obtain additional data. Their replies permitted the inclusion of the results to numerous survey questions. However, we were unable to obtain the results to question 2 by Kayapa et al. [18] and the results to question 1c by McClellan et al. [19].

Step 4. Consider whether each synthesis is likely to be biased because of the missing results in the studies identified.

We reported all 'known unknown' items in Appendix Table A39.

Step 5. Consider whether results from additional studies are likely to be missing from each synthesis: the 'unknown unknowns'.

We have contacted the authors of 2 surveys [20,21] (Figure 1)(Additional item J) to obtain the survey questions to assess the eligibility of these surveys, but after multiple attempts we did not receive a reply. We do not expect that many additional studies are likely to be missing from each synthesis, because we implemented broad spectrum search strategies and conducted searches in a wide body of data bases. Funnel plots to assess small study effects for all individual outcomes combined in the meta-analyses were not conducted, because there is no evidence on the validity of these graphs for proportional data [15].

Step 6. Reach an overall judgement about risk of bias due to missing results in each synthesis.

Our overall judgment is that the results to questions 1a, 1b, and 4 are at a moderate and the results to questions 1c and 2 at a high probability of risk of bias due to missing results in a synthesis (Appendix Table A39). The results of questions 1c and 2 had a higher probability of risk of bias, because for each of these questions the results of one survey, i.e., respectively those for the survey of Kayapa et al. [18] and those for the survey of McClellan et al. [19] were not given (Appendix Table A39).

Appendix Table A39. Risk of non-reporting bias for review items

Survey items and pertinent questions	Items that could have caused risk of bias due to missing results in a synthesis.	Overall judgment about risk of bias due to missing results in a synthesis
**Review item 1a.** Perceived honorary authorship of a co-author (without researchers being referred to any specific criteria for authorship).<br>**Question 1a.** Do you feel that any of your co-authors in this article did not make sufficient contributions to merit being included as co-authors?	• Unknown unknowns', i.e., risk of missing additional surveys	Moderate probability of risk of bias due to missing results in a synthesis
**Review item 1b.** Perceived honorary authorship based on ICMJE criteria.<br>**Question 1b**. As a result of your current understanding of ICMJE authorship guidelines, do you believe that any of your coauthors listed for this article did not make sufficient contributions to merit being included as coauthors?	• Unknown unknowns', i.e., risk of missing additional surveys	Moderate probability of risk of bias due to missing results in a synthesis
**Review item 1c.** Honorary authorship based on researchers' reported contributions of co-authors compared to the ICMJE criteria.<br>**Question 1c.** Did any of your co-authors perform only one or more of the following tasks, and nothing else, while working on this article?<br>These tasks refer to:<br>1. Supervising/recruiting co-authors 2. Obtaining funding or material support 3. Recruiting study subjects 4. Performing cases used in the study 5. Contributing illustrations 6. Reviewing the manuscript 7. Approving manuscript before submission to a journal 8. Signing statement of copyright transfer to journal	• 'Known unknowns', i.e., we were unable to obtain the results to Question 1c for one survey [19] with maximally 235 respondents.<br>• Unknown unknowns', i.e., risk of missing additional surveys	High probability of risk of bias due to missing results in a synthesis
**Review item 1c.** Honorary authorship based on researchers' reported contributions of co-authors compared to the ICMJE criteria.<br>**Question 1d.** How many of your coauthors had only one of the following functions, meaning they did only one of these functions and nothing else? (For each function below, give the number of coauthors, who did only that function)<br>These functions refer to:<br>1. Conceiving or designing the work 2. Conducting the literature search 3. Analyzing/interpreting literature/data 4. Performing statistical analysis 5. Writing the manuscript or part of the manuscript 6. Revising the manuscript critically for important intellectual content 7. Approving manuscript before submission to a journal 8. Supervising the work or any of the coauthors 9. Recruiting coauthors 10. Communicating with journal editor(s) 11. Obtaining funding or material support 12. Reviewing proofs or the journal's edited version of the review.	• Unknown unknowns', i.e., risk of missing additional surveys	Moderate probability of risk of bias due to missing results in a synthesis
**Review item 2.** Researchers having been approached by others to include honorary author(s) on a publication<br>**Question 2.** Did anyone suggest that you include an 'honorary' author in your manuscript?	• 'Known unknowns', i.e., we were unable to obtain the results to Question 1c for one survey [18] with 343 respondents<br>• Unknown unknowns', i.e., risk of missing additional surveys	High probability of risk of bias due to missing results in a synthesis
**Review item 3a**. Researchers admitting being an honorary author(s) on a publication based on a list of author's contributions. First author did not conceive or design the work, conduct literature search, or analyze and interpret data<br>**Question 3a.** Think about your role in the development of the review. Check all of the functions you personally performed for the review. Functions:<br>1. Conceiving or designing the work 2. Conducting the literature search 3. Analyzing/interpreting literature/data 4. Performing statistical analysis 5. Writing the manuscript or part of the manuscript 6. Revising the manuscript critically for important intellectual content 7. Approving manuscript before submission to a journal 8. Supervising the work or any of the coauthors 9. Recruiting coauthors 10. Communicating with journal editor(s)	• Unknown unknowns', i.e., risk of missing additional surveys	Moderate probability of risk of bias due to missing results in a synthesis

11. Obtaining funding or material support 12. Reviewing proofs or the journal's edited version of the review.		
**Review item 3b**. Researchers admitting being an honorary author(s) on a publication based on a list of author's contributions. First author did not draft or revise the review.<br>**Question 3b.** Think about your role in the development of the review. Check all of the functions you personally performed for the review. Functions:<br>1. Conceiving or designing the work 2. Conducting the literature search 3. Analyzing/interpreting literature/data 4. Performing statistical analysis 5. Writing the manuscript or part of the manuscript 6. Revising the manuscript critically for important intellectual content 7. Approving manuscript before submission to a journal 8. Supervising the work or any of the coauthors 9. Recruiting coauthors 10. Communicating with journal editor(s) 11. Obtaining funding or material support 12. Reviewing proofs or the journal's edited version of the review.	• Unknown unknowns', i.e., risk of missing additional surveys	Moderate probability of risk of bias due to missing results in a synthesis
**Review item 3c**. Researchers admitting being an honorary author(s) on a publication based on a list of author's contributions. First author did not give the final approval.<br>**Question 3c.** Think about your role in the development of the review. Check all of the functions you personally performed for the review. Functions:<br>1. Conceiving or designing the work 2. Conducting the literature search 3. Analyzing/interpreting literature/data 4. Performing statistical analysis 5. Writing the manuscript or part of the manuscript 6. Revising the manuscript critically for important intellectual content 7. Approving manuscript before submission to a journal 8. Supervising the work or any of the coauthors 9. Recruiting coauthors 10. Communicating with journal editor(s) 11. Obtaining funding or material support 12. Reviewing proofs or the journal's edited version of the review.	• Unknown unknowns', i.e., risk of missing additional surveys	Moderate probability of risk of bias due to missing results in a synthesis
**Review item 3d**. Researchers admitting being an honorary author(s) on a publication based on a list of author's contributions.<br>**Question 3d.** In the spaces marked "Contribution Codes," those code letters from the box should be marked that designate substantive contribution(s) of individual authors to the paper. These contribution codes refer to:<br>1. Conception and design 2. Analysis and interpretation of the data 3. Provision of study materials or patients 4. Collection, assembly and possession of raw data 5. Statistical expertise 6. Drafting of the article 7. Critical revision of the article for important intellectual content 8. Final approval of the article 9. Obtaining of funding 10. Administrative, technical, or logistic support 11. Guarantor of the study 12. Other (specify):	• Unknown unknowns', i.e., risk of missing additional surveys	Moderate probability of risk of bias due to missing results in a synthesis
**Review item 4.** Researchers (First authors) admitting adding an honorary author(s) on a publication.<br>**Question 4a.** Did you (First author) include an honorary author in your manuscript?	• Unknown unknowns', i.e., risk of missing additional surveys	Moderate probability of risk of bias due to missing results in a synthesis
**Review item 4.** Researchers (Senior authors) admitting adding an honorary author(s) on a publication.<br>**Question 4b**. Did you (Senior author, i.e., last author) include an honorary author in your manuscript?	• Unknown unknowns', i.e., risk of missing additional surveys	Moderate probability of risk of bias due to missing results in a synthesis
**Review item 5:** Researchers admitting having approached others to include honorary author(s) on a publication	Was not assessed	

Additional item O. Certainty of the evidence

Appendix Table A40. Summary of findings. Prevalence of honorary authorship issues in a publication on which the survyee was surveyed

Surveyee: Any author on the author list of a scientific publication, e.g., first, last, corresponding author, that was invited to participate in a survey on at least one of our review items. **Settings:** Any.

Review items	Prevalence	# of respondents and surveys	Certainty of the evidence (GRADE)*	Comments
**Review item 1a (Question 1a).** Perceived honorary authorship of a co-author (without researchers being referred to any specific criteria for authorship).	26%<br>[95% CI 21-31]	2,758<br>respondents<br>6 surveys	⊕⊖⊖⊖<br>**Very low**[a]<br>Due to risk of bias, inconsistency, imprecision, and moderate risk of non-reporting biases	Evidence from 6 surveys
**Review item 1b (Question 1b).** Perceived honorary authorship based on ICMJE criteria.	18%<br>[95% CI 15-21]	4,272<br>respondents<br>11 surveys	⊕⊖⊖⊖<br>**Very low**[b]<br>Due to risk of bias, inconsistency, imprecision, and moderate risk of non-reporting biases	Evidence from 10 surveys
**Review item 1c (Question 1c). **** Honorary authorship based on researchers' reported contributions of co-authors compared to the ICMJE criteria.	51%<br>[95% CI, 47-56]	5,111<br>respondents<br>15 surveys	⊕⊖⊖⊖<br>**Very low**[c]<br>Due to risk of bias, inconsistency, imprecision, and high risk of non-reporting biases	Evidence from 15 surveys<br>For this outcome the results of one survey [19] with maximally 235 respondents were not available
**Review item 1c (Question 1d).** ** Honorary authorship based on researchers' reported contributions of co-authors compared to the ICMJE criteria	22.2%<br>(148/666)<br>[95% CI, 19-26]	666 respondents<br>1 survey [22]	⊕⊕⊖⊖<br>**Low**[d]<br>Due to risk of bias, imprecision, and moderate risk of non-reporting biases	Evidence from 1 survey [22].<br>The results to question 1D could not be included in the meta-analysis for question 1c, because of different questions and tasks
**Review item 2 (Question 2).** ***Researchers having been approached by others to include honorary author(s) on a publication	10%<br>[95% CI, 9-12]	3,663<br>respondents<br>11 surveys	⊕⊖⊖⊖<br>**Very low**[e]<br>Due to risk of bias, inconsistency, and high risk of non-reporting biases	Evidence from 11 surveys<br>For this outcome the results of one survey [18] with 343 respondents were not available
**Review item 3a (Question 3a).** ** Researchers admitting being an honorary author(s) on a publication based on a list of author's contributions. Not contributed: First author did not conceive or design the work, conduct literature search, or analyze and interpret data	0.15% (1/666)<br>(95% CI 0.0038-0.83]	666 respondents<br>1 survey [22]	⊕⊕⊖⊖<br>**Low**[f]<br>Due to risk of bias, imprecision, and moderate risk of non-reporting biases	Evidence from 1 survey [22]
**Review item 3b (Question 3b)**. ** Researchers admitting being an honorary author(s) on a publication based on a list of author's contributions. Not contributed: First author did not draft or revise the review.	0.15% (1/666)<br>[95% CI 0.0038-0.83]	666 respondents<br>1 survey [22]	⊕⊕⊖⊖<br>**Low**[g]<br>Due to risk of bias, imprecision, and moderate risk of non-reporting biases	Evidence from 1 survey [22]
**Review item 3c (Question 3c).** ** Researchers admitting being an honorary author(s) on a publication based on a list of author's contributions. Not contributed: First author did not give the final approval.	7.2% (48/666)<br>(95% CI 5.4-9.4]	666 respondents<br>1 survey [22]	⊕⊕⊖⊖<br>**Low**[h]<br>Due to risk of bias, imprecision, and moderate risk of non-reporting biases	Evidence from 1 survey [22]
**Review item 3d (Question 3d). **** Researchers admitting being an honorary author(s) on a publication based on a list of author's contributions.	33.3% (67/201)<br>[95% CI 26.9 - 40.3]	201 respondents<br>1 survey [17]	⊕⊖⊖<br>**Very low**[i]<br>Due to risk of bias, imprecision, and moderate risk of non-reporting biases	Evidence from 1 survey [17]
**Review item 4 (Question 4a). **** Researchers (first authors) admitting adding an honorary author(s) on a publication.	16%<br>[95% CI 13-18]	823 respondents<br>2 surveys	⊕⊕⊖⊖⊖<br>**low**[j]<br>Due to risk of bias, inconsistency, and moderate risk of non-reporting biases	Evidence from 2 surveys
**Review item 4 (Question 4b). **** Researchers (Senior, i.e., last authors) admitting adding an honorary author(s) on a publication.	11.5% (10/87)<br>[95% CI, 5.7 - 20.1]	87 respondents<br>[19]	⊕⊖⊖⊖<br>**Very low**[k]<br>Due to risk of bias, imprecision, and moderate risk of non-reporting biases	Evidence from one survey [19]

*The rationales for the certainty grades (GRADE) are given in the Appendix
** International Committee of Medical Journal Editors (ICMJE)-based honorary authorship
*** Not specified whether it was perceived or ICMJE-based honorary authorship or both

Explanations for GRADE ratings

[a]The risk of bias was high for this outcome, because the overall confidence in the results of the 6 included surveys was rated as either low or critically low. The low P value and large Chi^2 (Chi^2 = 42.25 (df = 5) P<0.001) provide evidence of heterogeneity and the high I^2 (I^2 = 86.16%) indicates considerable inconsistency across the prevalence statistics of the surveys. The wide confidence intervals indicate imprecision and there was moderate probability of non-reporting biases (Figure 2).

[b]The risk of bias was high for this outcome, because the overall confidence in the results of the 10 included surveys was rated as either low or critically low. The low P value and large Chi^2 (Chi^2 = 86.99 (df = 9) P<0.001) provide evidence of heterogeneity and the high I^2 (I^2 = 89.65%) indicates considerable inconsistency across the prevalence statistics of the surveys. The wide confidence intervals indicate imprecision and there was moderate probability of non-reporting biases (Figure 3).

[c]The risk of bias was high for this outcome, because the overall confidence in the results of the 15 included surveys was rated as either low or critically low. The low P value and large Chi^2 (Chi^2 = 147.74 (df = 14) P<0.001) provide evidence of heterogeneity and the high I^2 (I^2 = 90.52%) indicates considerable inconsistency across the prevalence statistics of the surveys. The wide confidence intervals indicate imprecision and there was high probability of non-reporting biases (Figure 4).

[d]Risk of bias was present for this outcome, because the overall confidence in the results of this survey was rated as low. Imprecision was present, i.e., small number of studies (or single study) and/or a small number of participants (per study). There was moderate probability of non-reporting biases.

[e]The risk of bias was high for this outcome, because the overall confidence in the results of the 11 included surveys was rated as either low or critically low. The low P value and large Chi^2 (Chi^2 = 21.61 (df = 10) P<0.001) provide evidence of heterogeneity and the I^2 of 54.16%) indicates substantial inconsistency across the prevalence statistics of the surveys. There was high probability of non-reporting biases (Figure 5). It was also not clear whether HA was perceived or ICMJE-based.

[f]Risk of bias was present for this outcome, because the overall confidence in the results of this survey was rated as low. Imprecision was present, i.e., small number of studies (or single study) and/or a small number of participants (per study). There was moderate probability of non-reporting biases.

[g]Risk of bias was present for this outcome, because the overall confidence in the results of this survey was rated as low. Imprecision was present, i.e., small number of studies (or single study) and/or a small number of participants (per study). There was moderate probability of non-reporting biases.

[h]Risk of bias was present for this outcome, because the overall confidence in the results of this survey was rated as 'low'. Imprecision was present, i.e., small number of studies (or single study) and/or a small number of participants (per study). There was moderate probability of non-reporting biases.

[i]The risk of bias was high for this outcome, because the overall confidence in the results of this survey was rated as low. Imprecision was present, i.e., small number of studies (or single study) and/or a small number of participants (per study). There was moderate probability of non-reporting biases.

[j]The risk of bias was high for this outcome, because the overall confidence in the results of the 2 included surveys was rated either as low or critically low. The magnitude of the effect sizes of the 2 included surveys provide evidence of heterogeneity. The wide confidence intervals indicate imprecision and there was moderate probability of non-reporting biases (Figure 6).

[k]The risk of bias was high for this outcome, because the overall confidence in the results of this survey was rated as critically low. Imprecision was present, i.e., small number of studies (or single study) and/or a small number of participants (per study). There was moderate probability of non-reporting biases.

Additional item P. References

1. Deeks JJ, Higgins JPT, Altman DG (editors). Chapter 10: Analysing data and undertaking meta-analyses. In: Higgins JPT, Thomas J, Chandler J, Cumpston M, Li T, Page MJ, Welch VA (editors). Cochrane Handbook for Systematic Reviews of Interventions version 6.2 (updated February 2021). Cochrane, 2021. Available from www.training.cochrane.org/handbook.
2. Röver C, Friede T. Double arcsine transform not appropriate for meta-analysis. Res Synth Methods. 2022 Sep;13(5):645-648. doi: 10.1002/jrsm.1591. Epub 2022 Jul 22. PMID: 35837800.
3. Schwarzer G, Chemaitelly H, Abu-Raddad LJ, Rücker G. Seriously misleading results using inverse of Freeman-Tukey double arcsine transformation in meta-analysis of single proportions. Res Synth Methods. 2019 Sep;10(3):476-483. doi: 10.1002/jrsm.1348. Epub 2019 Apr 23. PMID: 30945438; PMCID: PMC6767151
4. Moher D, Shamseer L, Clarke M, Ghersi D, Liberati A, Petticrew M, Shekelle P, Stewart LA: Preferred Reporting Items for Systematic Review and Meta-Analysis Protocols (PRISMA-P) 2015 statement. Syst Rev 2015;4(1):1.
5. Shamseer L, Moher D, Clarke M, Ghersi D, Liberati A Deceased, Petticrew M, Shekelle P, Stewart LA; the PRISMA-P Group: Preferred reporting items for systematic review and meta-analysis protocols (PRISMA-P) 2015: elaboration and explanation. BMJ 2015;349:g7647.
6. Van Epps H, Astudillo O, del Pozo Martín Y, Marsh J. The Sex and Gender Equity in Research (SAGER) guidelines: Implementation and checklist development. Eur Sci Ed. 2022;48:e86910. https://doi.org/10.3897/ese.2022.e86910.
7. Bethlehem J. Chapter 12: A checklist for polls. In 'Understanding Public Opinion Polls'. Boca Raton, Florida (USA): Chapman and Hall/CRC; 2017. ISBN: 978-1498769747.

8. Meursinge Reynders R, Ter Riet G, Di Girolamo N, Malički M. Honorary authorship in health sciences: a protocol for a systematic review of survey research. Syst Rev. 2022 Apr 4;11(1):57. doi: 10.1186/s13643-022-01928-1. PMID: 35379330; PMCID: PMC8978359.
9. Sergeant ESG. Sergeant, ESG, 2018. Epitools Epidemiological Calculators. Ausvet. [online] Available from: http://epitools.ausvet.com.au. (accessed August 10th 2023).
10. Shea BJ, Reeves BC, Wells G, Thuku M, Hamel C, Moran J, Moher D, Tugwell P, Welch V, Kristjansson E, Henry DA. AMSTAR 2: a critical appraisal tool for systematic reviews that include randomised or non randomised studies of healthcare interventions, or both. BMJ. 2017 Sep 21;358:j4008. doi: 10.1136/bmj.j4008.
11. McKenzie JE, Brennan SE, Ryan RE, Thomson HJ, Johnston RV. Chapter 9: Summarizing study characteristics and preparing for synthesis. In: Higgins JPT, Thomas J, Chandler J, Cumpston M, Li T, Page MJ, Welch VA (editors). Cochrane Handbook for Systematic Reviews of Interventions version 6.2 (updated February 2021). Cochrane, 2021. Available from www.training.cochrane.org/handbook.
12. McKenzie JE, Brennan SE. Chapter 12: Synthesizing and presenting findings using other methods. In: Higgins JPT, Thomas J, Chandler J, Cumpston M, Li T, Page MJ, Welch VA (editors). Cochrane Handbook for Systematic Reviews of Interventions version 6.2 (updated February 2021). Cochrane, 2021. Available from www.training.cochrane.org/handbook.
13. Borenstein M, Hedges LV, Higgins JPT, Rothstein HR: Chapter 20: Meta-regression. In Introduction to Meta-Analysis. Edited by Borenstein M, Hedges LV, Higgins JPT, Rothstein HR. Chichester (UK): John Wiley & Sons; 2009.
14. Page MJ, Higgins JPT, Sterne JAC. Chapter 13: Assessing risk of bias due to missing results in a synthesis. In: Higgins JPT, Thomas J, Chandler J, Cumpston M, Li T, Page MJ, Welch VA

(editors). *Cochrane Handbook for Systematic Reviews of Interventions* version 6.3 (updated February 2022). Cochrane, 2022. Available from www.training.cochrane.org/handbook.

15. Barker TH, Migliavaca CB, Stein C, Colpani V, Falavigna M, Aromataris E, Munn Z. Conducting proportional meta-analysis in different types of systematic reviews: a guide for synthesisers of evidence. BMC Med Res Methodol. 2021 Sep 20;21(1):189. doi: 10.1186/s12874-021-01381-z. PMID: 34544368; PMCID: PMC8451728.
16. Schünemann HJ, Higgins JPT, Vist GE, Glasziou P, Akl EA, Skoetz N, Guyatt GH. Chapter 14: Completing 'Summary of findings' tables and grading the certainty of the evidence. In: Higgins JPT, Thomas J, Chandler J, Cumpston M, Li T, Page MJ, Welch VA (editors). Cochrane Handbook for Systematic Reviews of Interventions version 6.2 (updated February 2021). Cochrane, 2021. Available from www.training.cochrane.org/handbook.
17. Ilakovac V, Fister K, Marusic M, Marusic A. Reliability of disclosure forms of authors' contributions. CMAJ. 2007 Jan 2;176(1):41-6. doi: 10.1503/cmaj.060687. PMID: 17200389; PMCID: PMC1764586.
18. Kayapa B, Jhingoer S, Nijsten T, Gadjradj PS. The prevalence of honorary authorship in the dermatological literature. *Br J Dermatol*. 2018;178(6):1464–1465. doi:10.1111/bjd.16678
19. McClellan JM, Mansukhani N, Moe D, Derickson M, Chiu S, Kibbe MR, Martin MJ. Courtesy Authorship in Academic Surgery Publications. JAMA Surg. 2019 Dec 1;154(12):1110-1116. doi: 10.1001/jamasurg.2019.3140. PMID: 31532464; PMCID: PMC6752091.
20. Hoen WP, Walvoort HC, Overbeke AJ. What are the factors determining authorship and the order of the authors' names? A study among authors of the Nederlands Tijdschrift voor Geneeskunde (Dutch Journal of Medicine). JAMA. 1998 Jul 15;280(3):217-8. doi: 10.1001/jama.280.3.217. PMID: 9676659.

21. Vinther S, Rosenberg J. Appearance of ghost and gift authors in Ugeskrift for Læger and Danish Medical Journal. Dan Med J. 2012 May;59(5):A4455. PMID: 22549492.
22. Gülen S, Fonnes S, Andresen K, Rosenberg J. More than one-third of Cochrane reviews had gift authors, whereas ghost authorship was rare. J Clin Epidemiol. 2020 Dec;128:13-19. doi: 10.1016/j.jclinepi.2020.08.004. Epub 2020 Aug 8. PMID: 32781115.

Additional item Q. Legends for the tables of the Appendix

Appendix Table A1. Inclusion and exclusion criteria

Appendix Table A2. Search strategies for PubMed, Lens.org, and Dimensions.ai

Appendix Table A3. Data collection forms at the study level in surveys on HA issues*

Appendix Table A4. Data collection forms at the eligibility level in surveys on HA issues*

Appendix Table A5. Data collection forms at the sampling level in surveys on HA issues*

Appendix Table A6. Data collection forms at the survey methods level in surveys on HA issues*

Appendix Table A7. Data collection forms at the surveyee level in surveys on HA issues*

Appendix Table A8. Data collection forms at the response rate level in surveys on HA issues*

Appendix Table A9. Data collection forms for review item 1*

Appendix Table A10. Data collection forms for review item 2*

Appendix Table A11. Quality checklist for results of surveys on review item (#)*

Appendix Table A12. Signaling questions for Item 5. Survey methods*

Appendix Table A13. Rating the overall confidence in the results of a survey*

Appendix Table A14. Tabular presentation of the scores of the 14-item quality checklist for review item (#)* **

Appendix Table A15. Definition of response rates and primary and secondary outcomes

Appendix Table A16. Included surveys

Appendix Table A17. Characteristics of included surveys

Appendix Table A18. Characteristics of included surveys

Appendix Table A19. Characteristics of included surveys

Appendix Table A20. Excluded studies with rationale

Appendix Table A21. Rating the overall confidence in the results of a survey*

Appendix Table A22. Results for Question 1a.

Appendix Table A23. Results for Question 1b.

Appendix Table A24. Results for Question 1c

Appendix Table A25. Results for Question 2.

Appendix Table A26. Results for Question 4a

Appendix Table A27. Results for Question 1d.

Appendix Table A28. Results for Question 3a.*

Appendix Table A29. Results for Question 3b.*

Appendix Table A30. Results for Question 3c.*

Appendix Table A31. Results for Question 3d.*

Appendix Table A32. Results for Question 4b.

Appendix Table A 33. Question 1b.

Appendix Table A34. Question 1c.

Appendix Table A35. Question 2.

Appendix Table A36. Question 1b.

Appendix Table A37. Question 1c.

Appendix Table A38. Question 2.

Appendix Table A39. Risk of non-reporting bias for review items

Appendix Table A40. Summary of findings. Prevalence of honorary authorship issues in a publication on which the survyee was surveyed
